# Effect of Restricting Access to Health Care on Health Expenditures among Asylum-Seekers and Refugees: A Quasi-Experimental Study in Germany, 1994–2013

**DOI:** 10.1371/journal.pone.0131483

**Published:** 2015-07-22

**Authors:** Kayvan Bozorgmehr, Oliver Razum

**Affiliations:** 1 Department of General Practice & Health Services Research, University Heidelberg, Heidelberg, Germany; 2 Department of Epidemiology & International Public Health, School of Public Health, Bielefeld University, Bielefeld, Germany; Public Health Agency of Barcelona, SPAIN

## Abstract

**Background:**

Access to health care for asylum-seekers and refugees (AS&R) in Germany is initially restricted before regular access is granted, allegedly leading to delayed care and increasing costs of care. We analyse the effects of (a) restricted access; and (b) two major policy reforms (1997, 2007) on incident health expenditures for AS&R in 1994-2013.

**Methods and Findings:**

We used annual, nation-wide, aggregate data of the German Federal Statistics Office (1994-2013) to compare incident health expenditures among AS&R with restricted access (exposed) to AS&R with regular access (unexposed). We calculated incidence rate differences (*∆IR_t_*) and rate ratios (*IRR_t_*), as well as attributable fractions among the exposed (*AFe*) and the total population (*AFp*). The effects of between-group differences in need, and of policy reforms, on differences in per capita expenditures were assessed in (segmented) linear regression models. The exposed and unexposed groups comprised 4.16 and 1.53 million person-years. Per capita expenditures (1994–2013) were higher in the group with restricted access in absolute (*∆IR_t_* = 375.80 Euros [375.77; 375.89]) and relative terms (*IRR* = 1.39). The *AFe* was 28.07% and the *AFp* 22.21%. Between-group differences in mean age and in the type of accommodation were the main independent predictors of between-group expenditure differences. Need variables explained 50-75% of the variation in between-group differences over time. The 1997 policy reform significantly increased *∆IR_t_* adjusted for secular trends and between-group differences in age (by 600.0 Euros [212.6; 986.2]) and sex (by 867.0 Euros [390.9; 1342.5]). The 2007 policy reform had no such effect.

**Conclusion:**

The cost of excluding AS&R from health care appears ultimately higher than granting regular access to care. Excess expenditures attributable to the restriction were substantial and could not be completely explained by differences in need. An evidence-informed discourse on access to health care for AS&R in Germany is needed; it urgently requires high-quality, individual-level data.

## Introduction

Germany is one of ten countries in the European Union in which access to health care for asylum-seekers and refugees (AS&R) entering the country is initially restricted [1]. The “restrictionism” [2] in German asylum policy roots back to the early 1990s and aims at reducing the (alleged) abuse of the right to asylum. The Asylum-Seekers’ Benefits Act (*Asylbewerberleistungsgesetz*, *AsylbLG*) of 1 November 1993 separated welfare provisions for AS&R from the general welfare system. Since then, the Act has regulated the eligibility for and level of coverage with benefits related to existential human needs (such as housing, food, clothing and health care), which are provided on a minimum or substandard level [3].

### Entitlements, restrictions and conditions for regular access to health care

**Fig 1:**
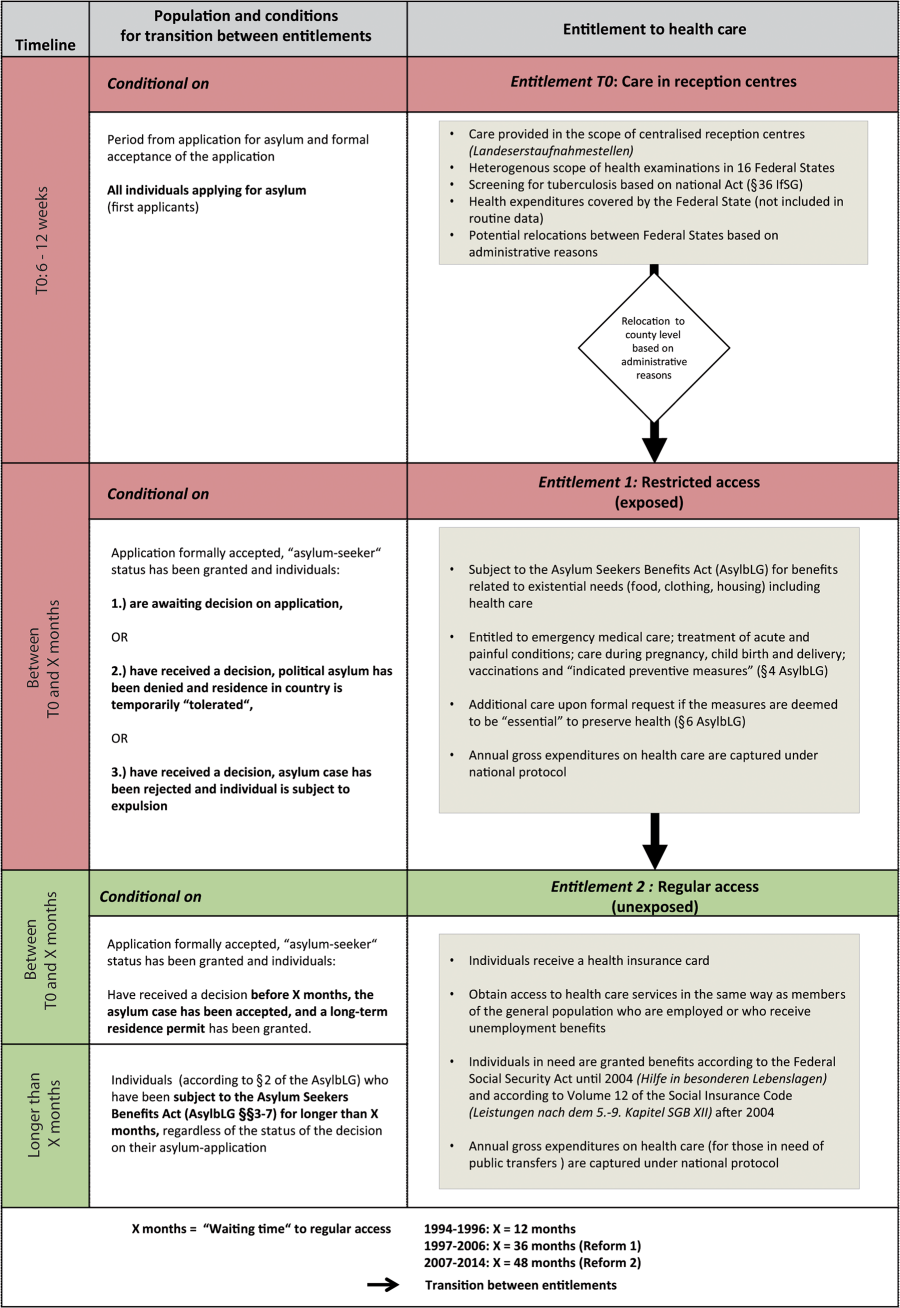
Simplified overview of entitlements to health care among asylum-seekers and refugees in Germany and transition between entitlements conditional on time and residence status.

AS&R fall into different categories of entitlement (Fig 1) depending on (i) their legal residence status and (ii) a “waiting time” regulation [1,3,4]. Restricted access is granted to individuals awaiting decisions on their asylum case (first or subsequent applicants); to refugees who are denied political asylum but cannot be repatriated on various grounds; to individuals whose asylum case has been rejected and who are subject to expulsion; as well as to asylum-seekers who have been granted a temporary residence permit on humanitarian grounds. These groups are entitled to emergency medical care, treatment for acute and painful conditions, care during pregnancy and childbirth, vaccinations and other “necessary preventive measures” (AsylbLG §4). Additional care may be granted upon formal request if the measures are deemed to be “essential” to preserve health (AsylbLG §6).

These legal restrictions are coupled with administrative barriers: service providers grant access to any type of ambulatory or specialist care only to AS&R who hold a valid healthcare-voucher (except for emergencies and for health care provided in reception centres). These healthcare-vouchers are valid for a limited period of time (e.g. three months). They are required to access any type of health care service and are a substitute for insurance cards used by the general population who are members of a Statutory Sickness Fund. To obtain a healthcare-voucher, AS&R must make a personal request at the local welfare agency, sometimes several times at different agencies, and repeatedly for every subsequent visit to a service provider.

A change in entitlement regarding access to health care and other welfare benefits, i.e. a shift from restricted to regular access to respective services, is conditional on a change in residence status or on the duration of time spent in Germany under restricted access. Regular access to health services treatment is granted to asylum-seekers who have been granted both asylum and a long-term residence permit, and according to section 2 of the Act (AsylbLG §2) to those whose case has not yet been decided, but who have received restricted benefits according to the Asylum-Seekers’ Benefits Act for a certain period of time, here referred to as “waiting time” (Fig 1).

AS&R entitled to regular access receive a health insurance card and thus obtain access to health care services and to other welfare benefits in the same way as members of the general population who are employed or receive unemployment benefits. The “waiting time” to regular access to health care (and to other welfare benefits) has been subject to several restrictive amendments since 1993 [2,3]: the first amendment (1 June 1997) prolonged the “waiting time” from 12 to 36 months, a second amendment (28 August 2007) to 48 months.

### Entitlements, restrictions and costs of health care

Entitlements on access to health care and other existential welfare services are important post-migration factors with the potential to affect not only health care needs among AS&R [5,6], but also important health system goals, such as equity, efficiency, quality and outcomes of care [7]. The restrictions, have been imposed with the rationale to safeguard public money [2,4]. It is possible, however, that providing existential services on a minimum level achieves exactly the opposite.

#### Direct effects of restrictive policies on health care costs

The legal restrictions on access to health care and the administrative barriers in Germany have been criticised since the 1990s [4] for leading to delayed care, for increasing direct costs and administrative costs of health care, and for shifting the responsibility for care from the less expensive primary care sector to costly treatments for acute conditions in the secondary and tertiary sector.

#### Indirect effects mediated by post-migration social determinants of health

Beyond these potential direct effects on health care costs, there are several links between legal entitlements and health care costs that might be mediated through differences in the social determinants of health [8] between the group with restricted and regular access.

With few exceptions, AS&R subject to the Asylum-Seekers’ Benefits Act who have not yet passed the “waiting time” (Fig 1) must reside in institutional facilities, i.e. in collective accommodations with shared sanitary facilities, rooms and kitchens. The geographical location of facilities, often barracks or camps outside city centres, may reduce geographical accessibility to needed services [9] and potentially exacerbate the delay in care “caused” by legal and administrative regulations.

#### Indirect effects mediated by post-migration social determinants of health and need components

The type of housing may further affect need components: AS&R residing in institutional facilities are at risk of having a higher burden of mental health problems compared to those in non-institutional accommodations [10]. Crowded institutional accommodations, in combination with inadequate immunization programmes, repeatedly lead to outbreaks of vaccine-preventable diseases among AS&R in Germany [11–13], causing higher costs for containment strategies than for the provision of full vaccine coverage [13]. Furthermore, during the initial year of their stay in the country or longer, AS&R must often undergo frequent relocations between institutional facilities which is a risk factor for mental distress among children [14].

Differences in entitlements to welfare benefits within the population of AS&R directly affect important social determinants of health such as income to meet daily needs: AS&R subject to restrictions imposed by the Asylum-Seekers Benefits Act have (until recently [15]) been granted a minimum level of financial benefits, partly replaced by benefits in kind, to meet existential needs [2,3]. The level of coverage with social transfer-payments for AS&R entitled to restricted access to health care has not changed between 1993 and 2013, and has been 30% to 47% lower than those granted to “normal” citizens in need [16]. AS&R with regular access, on the other hand, are granted social transfer-payments on the same level as individuals in need in the general population. Further differences within the population of AS&R refer to the right to enter the labour market, which not only affects income, but also psychosocial well-being [17,18], and thus health.

#### Indirect effects mediated through psychosocial factors and embodiment

The above restrictions can be conceptualised as part of an “othering” process, understood as “a process of marginalisation, disempowerment and social exclusion” [19], constantly reminding AS&R who are subject to those policies of being alien and sub-ordinate [18] to the general population. Actual and perceived discriminations may lead to psychosocial and physical morbidity [20] e.g. through embodiment processes [21], increasing needs among AS&R subject to imposed restrictions.

Despite the plausible direct and indirect links between different entitlements (to existential welfare benefits including health care (Fig 1)) and health care costs, no empirical evidence has been established yet for this relationship among AS&R in Germany or other countries in Europe. Civil society organisations (CSOs) in Germany argue that the costs of health care for AS&R with restricted access have always been higher than among those with regular access [22].

The validity of these claims has not yet been rigorously scrutinised with respect to the full time period in which restrictive policies have been in force (1994–2013). It has also not been considered yet that potential differences in costs of health care between AS&R with different entitlements may be attributable to differences in predisposing socio-demographic factors (such as sex and age) [9], as well as to differences in migration-related factors: different groups may have been exposed to different pre- and peri-migration health risks, and thus may bring along different needs for health care depending on the burden of disease in their country of origin and migration routes [23]. These factors may affect the need for health care in the population of AS&R. We therefore aimed to:

1. examine the effects of restricted entitlements to health care on incident health expenditures for asylum-seekers and refugees (AS&R) in Germany between 1994 and 2013,
2. analyse if differences in per capita health expenditure between the two groups (restricted vs. regular access) can be explained by differences in underlying needs,
3. evaluate the effect of two major policy amendments during the observation period on expenditure differences between the two groups (restricted vs. regular access).

## Methods

### Study design and observation period

The fact that AS&R in Germany are entitled to different types of health care and welfare benefits depending on their legal residence status and the “waiting time” regulation (Fig 1) leads to a situation resembling the design of a quasi-experimental study, which we exploited for achieving study objectives 1 and 2. The policy amendments of 1997 and 2007, which further restricted access to health care among AS&R, allowed constructing a historically prospective interrupted time series starting in 1994 to achieve objective 3.

### Data sources and variables

We obtained data from the Federal Statistics Office (FSO) on all AS&R registered in Germany between 1994 and 2013. In the scope of a national protocol, the FSO captures two types of data that were used for this study:

1. Census data on the total number of AS&R registered in Germany at the end of each year, including information on age, sex, residence status, entitlement to benefits according to the AsylbLG, country and continent of origin, and type of accommodation (institutional vs. non-institutional/private).
2. Data on gross annual expenditures on different types of benefits (including health care) according to the AsylbLG.

Data are collected by the local authorities at municipality level, reported to the statistics offices at federal state level, aggregated at national level by the FSO and reported as count data. No individual-level data is publicly available to ensure data protection. All analyses performed were thus built upon ecological (aggregate) data.

### Exposure status

We defined the population specified in section 1 of the Asylum Seekers’ Benefits Act (AsylbLG §1), reported by the FSO as *“Grundleistungsempfänger”*, as “exposed” to restricted access (according to AsylbLG §§4,6). The population of AS&R that was subject to section 2 of the Act (AsylbLG §2), and was as such entitled to regular access to health care analogously to the general population at 31 December of each year (*“EmpfängerInnen von Hilfe zum Lebensunterhalt”*), was defined as “unexposed”.

### Outcome

The outcome of interest were differences in incident health expenditures (in Euro) for AS&R in each group during the observation period (1994–2013). We calculated incidence rates at measurement occasion *t* (*IR*_*t*_ *)* for each group (exposed and unexposed) as:

$$IR_{t}\;=\frac{THE_{t}}{N_{t}},$$ (Eq 1)

where *THE* is total expenditure on health among each group, divided by the respective population at risk (*N*) in each group and year (i.e. divided by the total number of AS&R in each group registered on 31 December, 1994–2013). Assuming that *N*_*t*_ was fix throughout the year for both exposed and unexposed, *IR*_*t*_ can be interpreted as per capita expenditure on health (in Euro) at measurement occasion *t* among exposed/unexposed. Details on the types of expenditure used to calculate *THE* among each group is provided in Table 1 and the S1 Appendix.

**Table 1:** Type of officially reported expenditures used to calculate health expenditures among asylum-seekers with restricted and regular access to health care.

Health expenditures on asylum-seekers with		
	restricted access (exposed)	regular access (unexposed)
**Defined as**	◾ The sum of annual gross expenditures for services according to section 4 (*Leistungen bei Krankheit*, *Schwangerschaft und Geburt*, *AsylbLG §4)* and section 6 (*sonstige Leistungen*, *AsylbLG §6*) of the Act.	◾ The sum of annual gross expenditures for services according to the Federal Social Security Act until 2004 (*Hilfe in besonderen Lebenslagen*), and services according to Volume 12 of the Social Insurance Code (*Leistungen nach dem 5*.*-9*. *Kapitel SGB XII*) 2005 onwards.
**Consist of**	◾ Expenditures for in-patient and out-patient treatment of acute or painful conditions (including dental care), vaccination, and preventive maternal care services including costs of delivery.	◾ Expenditures for the treatment of all conditions throughout the year (in-patient and out-patient care), i.e. for any services for which the Statutory Sickness Funds were re-imbursed by the Welfare Agencies.
**Include**	◾ Expenditures of other conditions (categorised under section 6) and may also include costs for medical aids, nursing support, or benefits in kind.	◾ Expenditures for several social service not related to health care such long-term care, medical aids, disability related costs of integration etc.
**Not included**	◾ Health care expenditures in reception centres (*Landeserstaufnahmestellen*) during the first 6–12 weeks of the asylum process are not included.	◾ Monthly premium-payments of Welfare Agencies to the Statutory Sickness Funds are not included, i.e. the health care-related expenditures exclusively consist of expenditures for treatment.
		◾ Health care-related expenditures on asylum-seekers who have been granted asylum *and* a long-term residence permit *and* who are (temporarily) unemployed are not included.

### Confounders and mediators

Our analysis was guided by a causal diagram () representing the pathways outlined in the introduction. We treated predisposing socio-demographic factors as well as pre- and peri-migration factors as potential confounders; these factors may be associated with differences in per capita health expenditure (*IR*_*t*_) between exposed and unexposed AS&R without necessarily being attributable to differences in entitlements. We used the continent of origin (percentage of AS&R with European, Asian, African, or American origin) and a residual category (percentage of AS&R with other/unknown continent of origin) to approximate pre- and peri-migration exposures to health risks in each group. Socio-demographic variables used to approximate predisposing need were mean age (aggregate group mean reported by the FSO) and percentage of women in each group.

**Fig 2:**
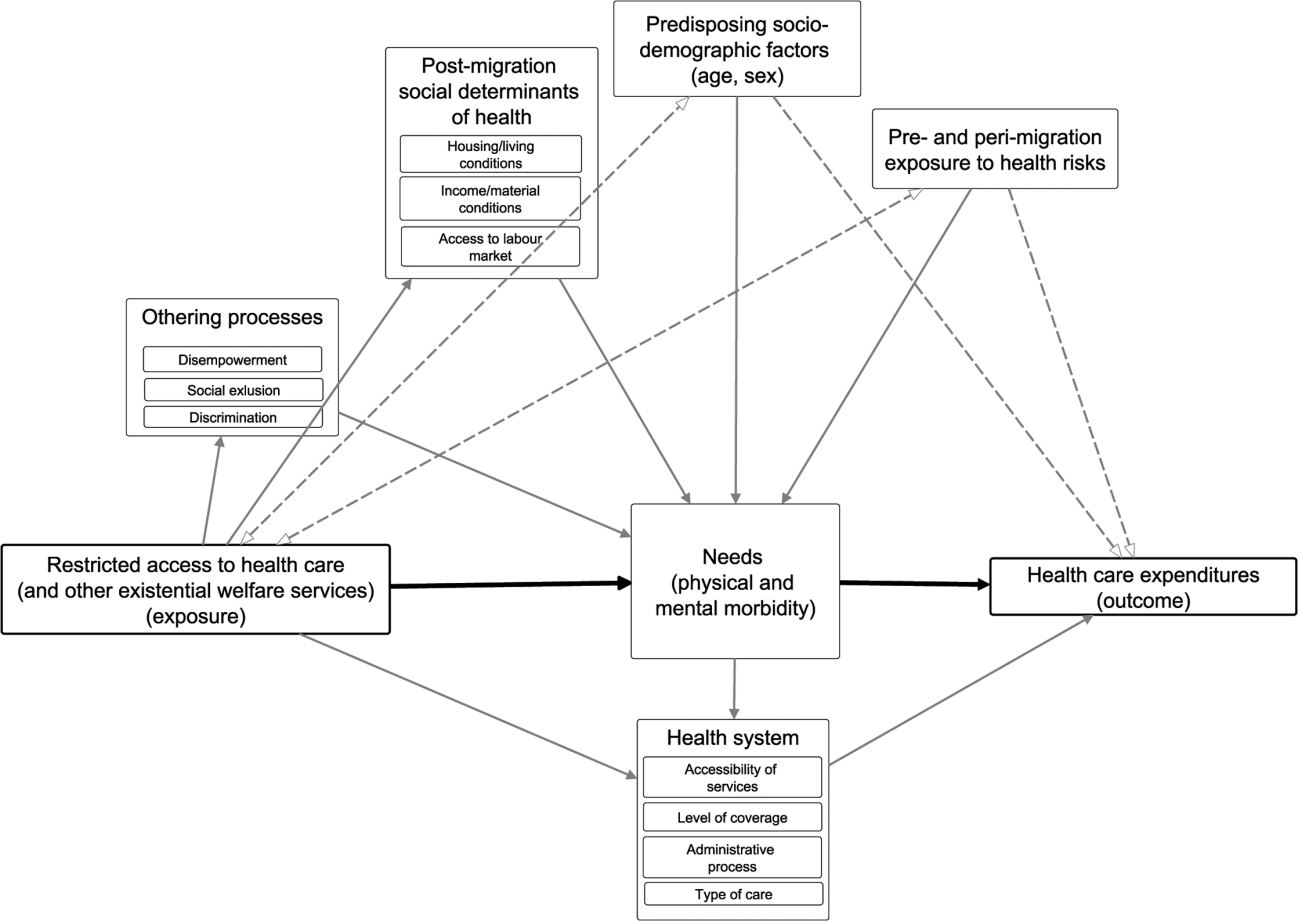
Causal diagram of the hypothetical relationship between restricted access to health care and health care expenditures including mediators and confounders of the association. Bold lines: mediating relations. Dashed lines: confounding relations. Causal relations: one-sided arrows. Non-causal relations: two-sided (hollow) arrows.

We conceptualised factors that are associated with different entitlements and may exert influence on health care costs through post-migration social determinants and morbidity as mediators (Fig 2) of the relationship between entitlements and costs. We used the percentage of AS&R living in non-institutional accommodation (i.e. decentralised apartments in municipalities with private sanitary facilities, rooms and kitchens) as a proxy for post-migration exposure to psychosocial and physical health risks. No other information on post-migration social determinants was available in data reported by the FSO.

Since all of the above factors are hypothesised to exert influence on costs through effects on need, we refer to all of them as “need variables” for ease of reading. Absolute differences in underlying need variables at measurement occasion *t* (∆NEED_*t*_) where calculated as:

$$\Delta NEED_{t}=NEED_{t,\text{exp}\;osed}-NEED_{t,un\;\text{exp}\;osed},$$ (Eq 2)

where *NEED* refers to the respective need variables (age, sex, continent of origin, type of accommodation) among exposed and unexposed. All information on confounders (except for age) was reported by the FSO as count data. We transformed count data to proportions using the denominators (*N*_*t*_) reported for each group.

### Statistical analysis

#### Effects of restricted access to health care on incident health expenditures (objective 1)

To examine the effects of access restrictions on the outcome (at measurement occasion *t*) we calculated (i) incidence rate differences (Δ*IR*_*t*_ = *IR*_*t*,exp *osed*_ − *IR*_*t*,*un* exp *osed*_) and rate ratios (IRRt=IRt,exposedIRt,unexposed), as well as (ii) attributable fractions among the exposed (*AFe*) and among the total population (*AFp*) (S1 Appendix) with respective 95% CIs. These measures were calculated for each year as well as for the whole observation period (1994–2013).

#### Differences in per capita health expenditure and relationship with need (objective 2)

We first assessed if there is a significant difference in the period mean (1994–2013) of per capita health expenditure (*IR*_*t*_), as well as in the period mean of underlying need variables between exposed and unexposed by means of t-tests for paired samples.

To analyse if absolute differences in per capita health expenditure between exposed and unexposed at measurement occasion t (*∆IR*_*t*_) could be explained by differences in underlying need variables (∆*NEED*_*t*_), we performed a generalized least square (GLS) linear regression analysis (Prais-Winsten-Regression) correcting for the presence of serial autocorrelation (type 1) and secular trends. The equation of the multiple regression model can be written as:

$$\Delta IR_{t}=\beta_{0}+\beta_{1}*TIME+\beta_{n}*\Delta NEED_{n,t}+\varepsilon_{t}.$$ (Eq 3)

The outcome *∆IR*_*t*_ is the absolute difference in per capita expenditure on health (in Euros) among AS&R in each group (*IR*_*t*, *exposed*_—*IR*_*t*, *unexposed*_) at measurement occasion *t*; *β*_*0*_ is the mean intercept (i.e. the baseline level of outcome at the beginning of the observation period), and *ε*_*t*_ is an error term which is assumed to have a normal distribution with mean zero and variance θ. *TIME* is a continuous variable starting a the beginning of the observation period; ∆NEED_*t*_ is the difference in one or more *(n)* need variables between the two groups (exposed *minus* controls) at measurement occasion *t*. The same analysis was also performed for the *AFe* as outcome (S4 and S5 Tables).

*β*_*1*_ estimates the secular trend in outcome regardless of the differences in need; *β*_*2*_ estimates the average change in outcome per unit increase in *∆NEED* adjusted for underlying secular trends. The scale of *∆NEED* is “percentage-points” for all need variables except for age, where the difference is measured in years. A one-unit increase in *∆NEED* means, for example, a percentage-point *increase* among AS&R living in non-institutional accommodation *in the exposed group*, or a percentage-point *decrease* in AS&R living in non-institutional accommodation *in the unexposed group*. Both scenarios lead to an increase in *∆NEED*. Univariate analyses were performed including *either* time *or* one need variable respectively (S3 and S4 Tables) prior to building the multiple regression model.

We performed regression diagnostics to check the linearity assumptions and excluded outliers (S2 Appendix) that could affect the relationship between outcome and respective *∆NEED* variables. Variation inflation factors (VIF) were calculated for all models; variables with VIF>10 were excluded from the analysis to avoid multi-collinearity. We tested for non-stationary trends in our data using correlographs of the outcome (S2 Appendix). Standard errors were clustered by year to account for the non-independence of observations.

### Effect of policy amendments on expenditure differences (objective 3)

To evaluate the effect of policy amendments during the observation period on the outcome (*∆IR*_*t*_), we additionally performed a segmented GLS linear regression analysis (Prais-Winsten-Regression) according to the following equation:

$$\Delta IR_{t}=\beta_{0}+\beta_{1}*TIME+\beta_{2}*REFORM1/2+\beta_{3}*POSTTREND\;\left[+\beta_{n}*\Delta NEED_{n,t}\right]+\varepsilon_{t}.$$ (Eq 4)

Compared to the model described in Eq 3, we here additionally capture the effects on the outcome (*∆IR*_*t*_) of *REFORM1/2*, a dummy for each amendment. *POSTTREND* is a time-dependent variable specific for each post-reform period (coded zero until the onset of the respective reform and sequentially from 1 thereafter). In Eq 4, *β*_*1*_ estimates the secular trend in outcome regardless of the reform; *β*_*2*_ estimates the immediate effect of the reform, i.e. the average change in level in the outcome in the post-reform compared to the pre-reform period corrected for pre-existing trends; and *β*_*3*_ reflects the annual change in trend after the respective reform. This model was also extended to control for ∆*NEED*. All analyses were performed using Stata Version 12.1.

#### Missing data

**Fig 3:**
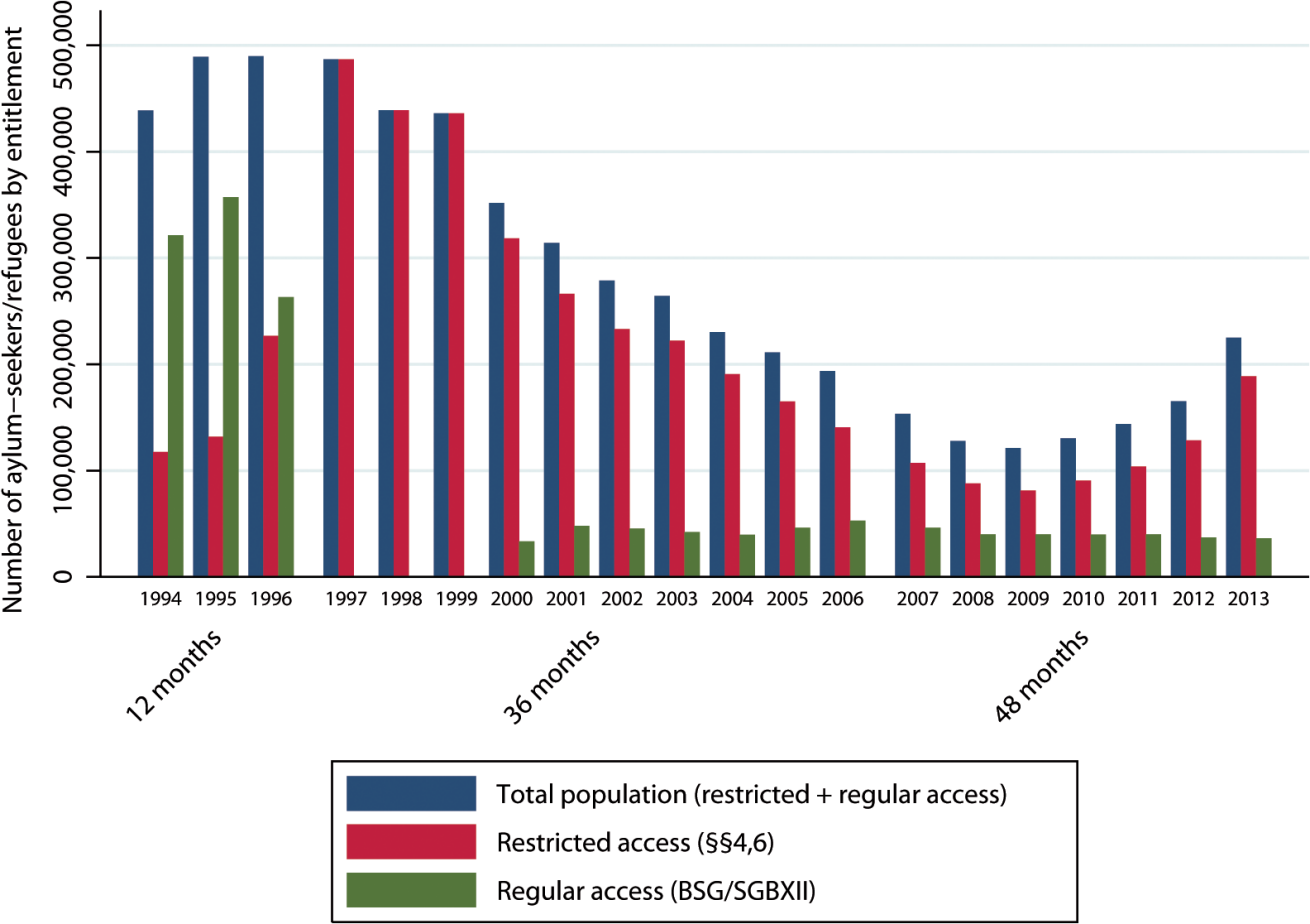
Population of asylum-seekers and refugees in Germany by entitlement of access to health care (1994–2013). Y-axis: shows the total number of asylum-seekers/refugees registered in Germany on 31 December of each year. Restricted access: refers to access to health care according to sections 4 and 6 of the Asylum-Seekers’ Benefits Act (AsylbLG §§4,6). Regular access: refers to access to health care analogously to the general population according to the Federal Social Security Act (Bundessozialhilfegesetz, BSG) before 2005 and to Volume 12 of the Social Insurance Code (*Leistungen nach dem 5*.*-9*. *Kapitel SGB XII*) thereafter. 12/36/48 months: indicate the “waiting time” to regular access (according to section 2 of the Asylum-Seekers’ Benefits Act, AsylbLG §2) in respective time periods.

There were no missing data for outcome or need variables. The sample size of the group with regular access (unexposed) was zero between 31 December 1997 and 31 December 1999 due to the first restrictive policy amendment (of June 1997) which brought about a change in entitlements regarding access to health care. This amendment of the AsylbLG (reform1) legally “eliminated” the population entitled to regular access until May 2000 (Fig 3). Per capita health expenditures for the group with regular access (*IR*_*t*, *unexposed*_) could not be calculated for years with zero denominators (1997–1999). These observations were thus excluded from the analysis to avoid artificially high absolute differences in outcome (*∆IR*_*t*_).

## Results

### Descriptive results

During the observation period (1994–2013), the groups with regular and restricted access comprised 4,160,712 and 1,528,111 person-years respectively; absolute health care expenditures amounted to 5.570 billion Euros (restricted access) and 1.472 billion Euros (regular access). The number of AS&R peaked in the 1990s, decreased in both groups until 2009, and was on the rise thereafter in the exposed group (see Fig 3). A detailed description of the study population in terms of sex, mean age, type of accommodation and health expenditure by type of entitlement is provided in Table 2.

**Table 2:** Descriptive details of the study population by year and type of access.

	Restricted access (exposed)					Regular access (unexposed)				
Year	N*	Female n (%)	Mean age	Non-institutional housing n (%)	Health expenditure^a^ (in Mill. Euro)	N*	Female n (%)	Mean age	Non-institutional housing n (%)	Health expenditure^b^ (in Mill. Euro)
1994	117,429	40,165 (34.2)	23.0	54,968 (46.8)	205.0	321,189	138,918 (43.3)	23.0	259,649 (80.8)	233.8
1995	131,820	43,342 (32.9)	23.0	67,258 (51)	197.0	357,154	157,627 (44.1)	25.0	280,986 (78.7)	265.3
1996	226,580	91,954 (40.6)	24.0	144,455 (63.8)	221.0	263,162	110,200 (41.9)	23.0	189,002 (71.8)	270.6
1997	486,643	199,542 (41)	24.0	289,341 (59.5)	357.0	0	0 (0)	-	0 (0)	154.1
1998	438,873	175,780 (40.1)	23.0	259,180 (59.1)	463.0	0	0 (0)	-	0 (0)	-
1999	435,930	180,619 (41.4)	23.0	245,588 (56.3)	449.0	0	0 (0)	-	0 (0)	-
2000	318,238	132,211 (41.5)	23.0	164,433 (51.7)	424.0	33,404	15,213 (45.5)	24.0	23,058 (69)	18.15
2001	266,064	105,801 (39.8)	24.0	125,319 (47.1)	369.0	48,052	22,305 (46.4)	25.0	31,313 (65.2)	40.69
2002	233,143	90,932 (39)	24.0	106,493 (45.7)	334.0	45,449	21,574 (47.5)	25.0	30,185 (66.4)	40.29
2003	222,162	87,073 (39.2)	24.0	105,167 (47.3)	317.0	42,078	19,918 (47.3)	25.0	29,430 (69.9)	54.12
2004	190,535	76,067 (39.9)	25.0	90,165 (47.3)	296.0	39,613	18,810 (47.5)	26.0	28,105 (70.9)	32.96
2005	165,016	66,438 (40.3)	25.0	82,545 (50)	278.0	46,106	21,985 (47.7)	26.0	32,475 (70.4)	47.16
2006	140,650	56,993 (40.5)	25.0	73,639 (52.4)	248.0	52,912	25,245 (47.7)	26.0	37,279 (70.5)	57.90
2007	107,200	42,041 (39.2)	26.0	55,672 (51.9)	207.0	46,100	22,184 (48.1)	27.0	33,752 (73.2)	65.30
2008	87,894	33,277 (37.9)	26.0	43,461 (49.4)	169.0	39,971	19,471 (48.7)	28.0	30,218 (75.6)	57.20
2009	81,314	30,184 (37.1)	27.0	38,596 (47.5)	153.0	39,921	19,402 (48.6)	29.0	30,288 (75.9)	49.67
2010	90,546	33,720 (37.2)	26.0	39,436 (43.6)	170.0	39,751	19,786 (49.8)	30.0	29,929 (75.3)	55.85
2011	103,767	38,930 (37.5)	26.0	44,676 (43.1)	186.0	39,920	20,123 (50.4)	31.0	30,857 (77.3)	62.47
2012	128,284	47,741 (37.2)	26.0	53,657 (41.8)	225.0	36,960	18,099 (49)	31.0	27,459 (74.3)	60.31
2013	188,624	69,678 (36.9)	25.0	82,892 (43.9)	303.0	36,369	17,442 (48)	32.0	26,399 (72.6)	59.75

**Restricted access**: refers to access to health care according to sections 4 and 6 of the Asylum-Seekers’ Benefits Act (AsylbLG §§4,6).

**Regular access**: refers to access to health care analogously to the general population according to the Federal Social Security Act (Bundessozialhilfegesetz, BSG) before 2005 and to Volume 12 of the Social Insurance Code (*Leistungen nach dem 5*.*-9*. *Kapitel SGB XII*) thereafter.

* refers to the population in each group on Dec 31 of each year.

^**a**^The sum of annual gross expenditures for services according to section 4 (*Leistungen bei Krankheit*, *Schwangerschaft und Geburt*, *AsylbLG §4)* and section 6 (*sonstige Leistungen*, *AsylbLG §6*) of the Act.

^**b**^ The sum of annual gross expenditures for services according to the Federal Social Security Act until 2004 (*Hilfe in besonderen Lebenslagen*), and services according to Volume 12 of the Social Insurance Code (*Leistungen nach dem 5*.*-9*. *Kapitel SGB XII*) 2005 onwards.

**Fig 4:**
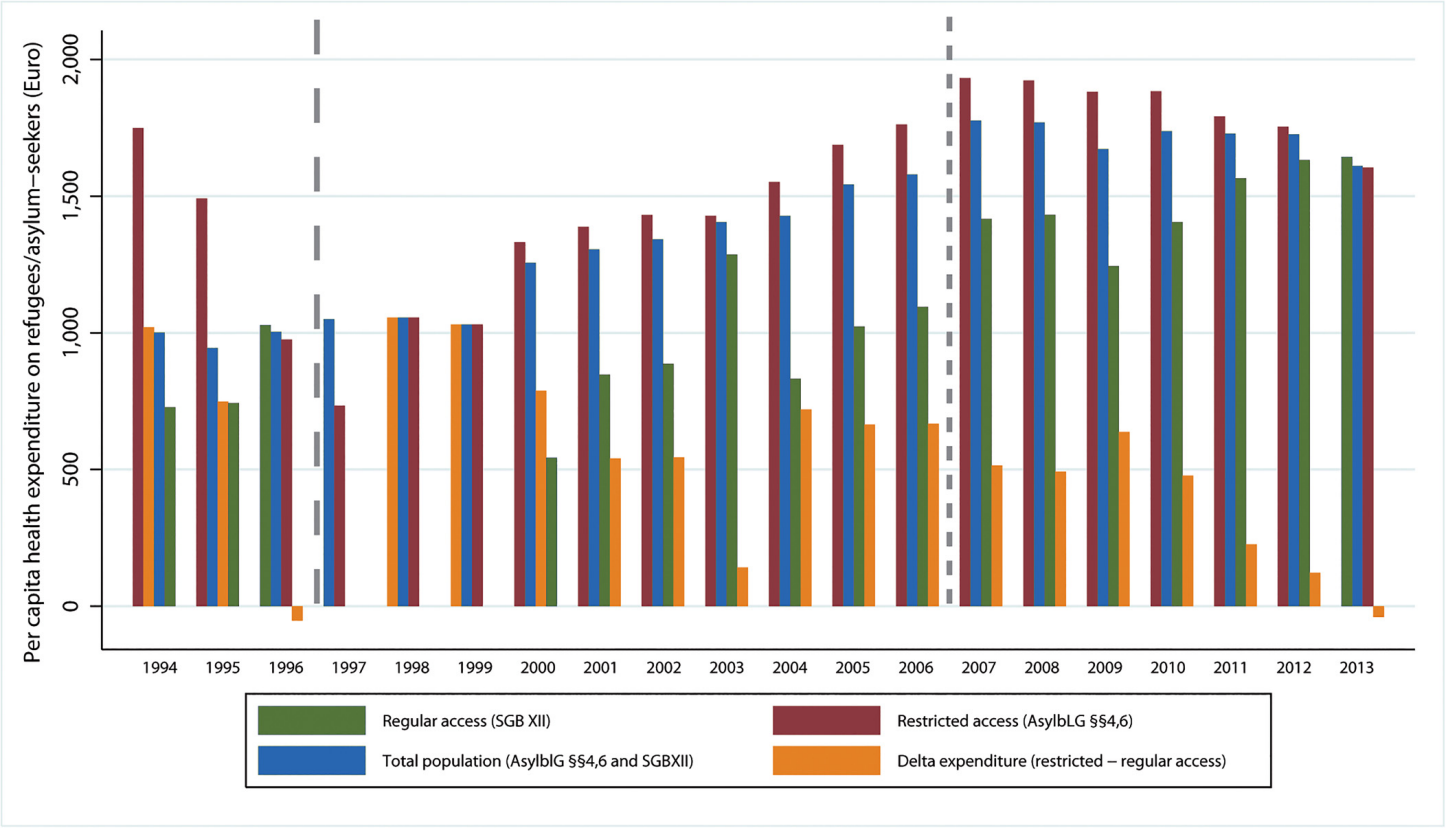
Per capita health expenditure on AS&R by type of access and absolute difference in per capita expenditure on health between the groups with restricted and regular access (1994–2013). Long-dashed vertical line: indicates onset of REFORM1 in June 1997, which prolonged the, “waiting time” to regular access from 12 months (1994–1996) to 36 months thereafter (until 2006). Short-dashed vertical line: indicates onset of REFORM2 in August 2007, which prolonged the, waiting time”to regular access from 36 months (1997–2006) to 48 months (2007–2013). The observations in 1997–1999 were excluded from the analysis because the group with regular access (on 31 Dec) was zero, thus leading to artificially high differences in expenditures, and in 1997 to artificially high per capita expenditures for the total population. Expenditures for regular access before 2005 refer to expenditures categorised under the Federal Social Security Act (*Bundessozialhilfegesetz*).

**Fig 5:**
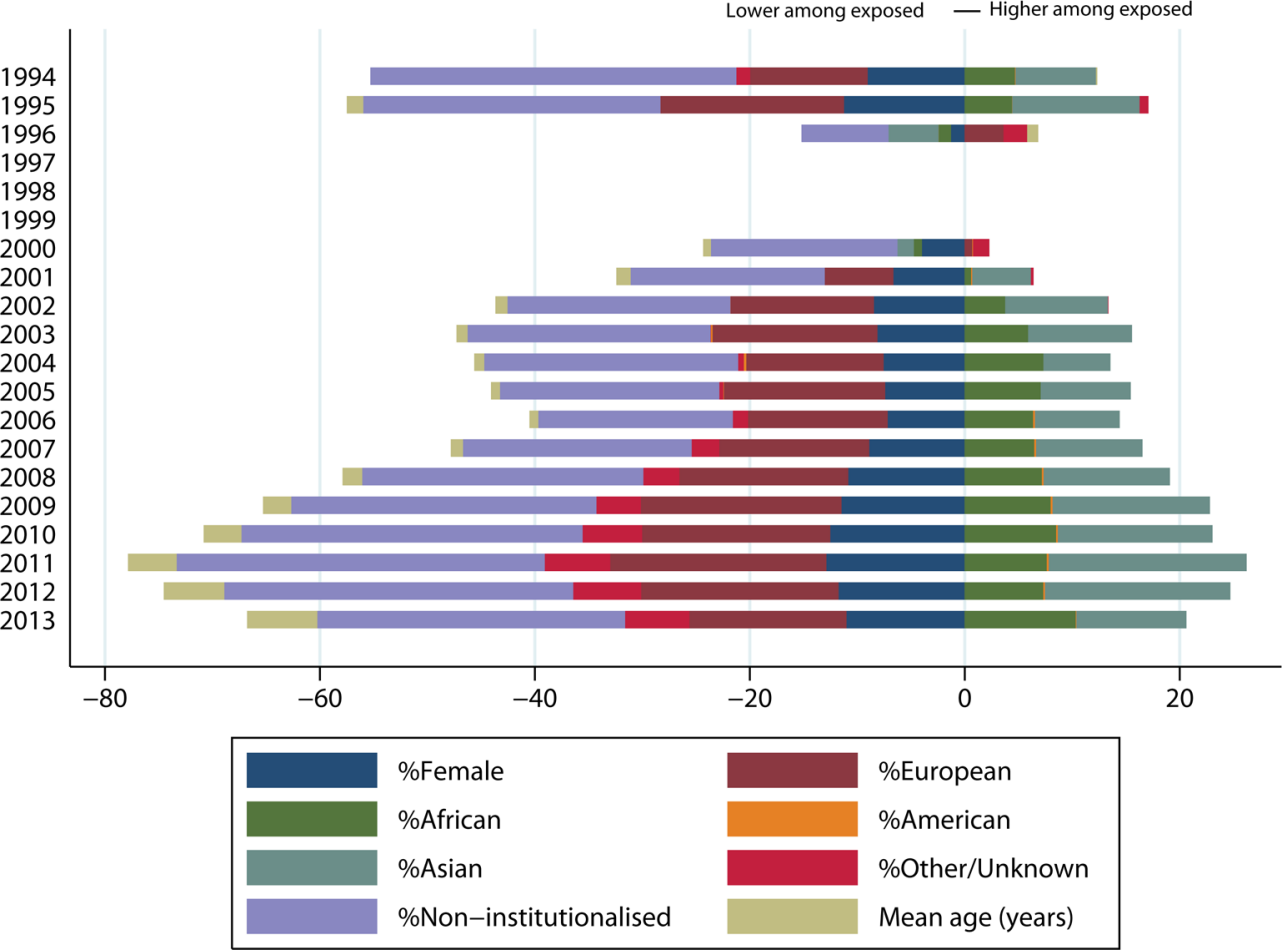
Absolute difference in need variables (exposed *minus* unexposed group). Y-axis: shows percentage-point differences between groups with restricted access (exposed) and regular access (unexposed) to health care for all need variables, except for, “mean age” where the difference is in years. The observations in 1997–1999 were excluded from the analysis because the group with regular access (on 31 Dec) was zero. The category, “Other/Unknown” comprises asylum-seekers with nationalities from Australia and Oceania, stateless asylum-seekers, and asylum-seekers for with unknown nationality.

Per capita health expenditures were higher throughout the whole observation period among the group with restricted access, except in 1996 and 2013 (Fig 4). The proportions of women, of individuals living in non-institutional accommodation, and of individuals with European nationality were lower in the exposed group (Fig 5). Details on the continents of origin of AS&R in each group are provided in the S1 Table.

### Effects of restricted access to health care on incident health expenditures

**Fig 6:**
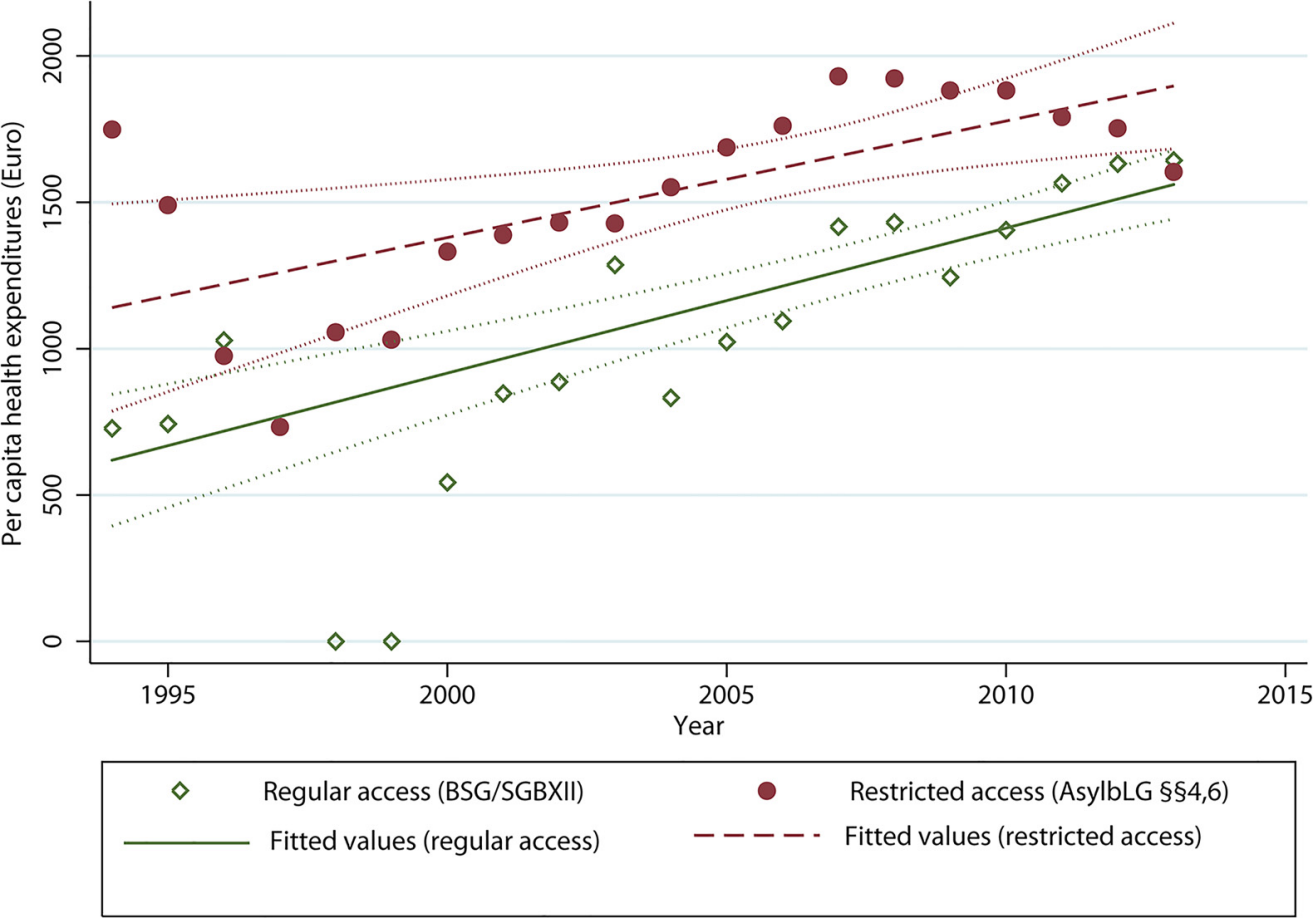
Scatter plot and fitted values of per capita health expenditures on asylum-seekers and refugees by entitlement on access to health care. Restricted access: refers to access to health care according to sections 4 and 6 of the Asylum-Seekers’ Benefits Act (AsylbLG §§4,6). Regular access: refers to access to health care analogously to the general population according to the Federal Social Security Act (Bundessozialhilfegesetz, BSG) before 2005 and to Volume 12 of the Social Insurance Code (*Leistungen nach dem 5*.*-9*. *Kapitel SGB XII*) thereafter. Dotted lines below/above fitted values: constitute 95% confidence intervals, obtained from robust standard errors clustered by year. The observations in 1997–1999 were excluded from the analysis in predicting fitted values for the group with regular access because the denominator (on 31 Dec) was zero.

The fitted values of per capita health expenditures among those with restricted access were significantly higher compared to the group with regular access, except for the beginning and end of the observation period, where expenditures (and 95% CIs) converged (Fig 6).

The incidence rate difference between groups with restricted and regular access for the whole period (1994–2013) was 375.80 Euros [375.77; 375.89], and the period *IRR* was 1.39 [1.3902; 1.3904]. During the two decades (1994–2013), the *AFe* was 28.072% [28.068; 28.077] and the *AFp* 22.21%. Over the two decades, this corresponds to expenses of 1.560 billion Euros that could have been averted in the absence of restricted access to health care and existential welfare services.

The annual incidence rate difference (*∆IR*_*t*_ *)* ranged between -52.87 Euros [-53.00; -52.65] in 1996 and +1020.6 Euros [1020.3; 1020.8] in 1994 (Fig 4). The annual IRRs ranged between 0.9486 [0.9480; 0.9488] in 1996 and 2.451 [2.449; 2.452] in the year 2000 (S1 Fig). The annual *AFe* ranged between 2.38% [2.35; 2.41] in 2013 and 59.19% [59.17; 59.21] in the year 2000, the *AFp* in these years ranged between 1.99% and 56.76% (S2 Fig).

### Differences in per capita health expenditure and relationship with need

The period mean of the outcome and the period mean of all need variables differed significantly (p<0.05) between the groups with restricted and regular access, except for the average proportion of AS&R with nationality from the American continents where no such difference (p = 0.137) could be found (S2 Table).

The GLS linear regression models (Table 3) showed that absolute differences in per capita health expenditure between AS&R with restricted and AS&R with regular access (*∆IR*_*t*_) could not be explained by differences in nationalities between exposed and unexposed, adjusted for secular trends as well as differences in needs variables (mean age and the proportion of women). Differences between the group with restricted access and the group with regular access in mean age and in the proportion of AS&R living in non-institutional accommodation turned out to be the main independent predictors of the differences in per capita health expenditure. A one-percentage-point increase in the proportion of AS&R in non-institutional accommodation in the group with restricted access reduced the difference in per capita health expenditures by 28.7 Euros, holding constant the differences (between exposed and unexposed) in mean age and in the proportion AS&R from different continents of origin (Table 3). Overall, 50–75% of the variation in *∆IR*_*t*_ over time could be explained by differences in *∆NEED* variables over time. The unadjusted crude results are shown in the S3 Table.

**Table 3:** Change in the dependent variable “absolute difference in per capita health expenditure (∆*IR*_*t*_)” in Euros per one unit increase in the independent variable ∆*NEED*_*t*_ (adjusted for secular trends and between-group differences in age and sex).

	M1	M2	M3	M4	M5	M6	M7	M8	M9
**Time** (years)	-35.14**	-14.69	-7.03	-4.006	-14.3	-11.35	-6.057	-34.04*	- excluded
	**[-54.94,-15.35]**	[-30.15,0.765]	[-23.92,9.856]	[-26.15,18.14]	[-36.01,7.419]	[-40.51,17.80]	[-25.45,13.34]	**[-65.34,-2.741]**	
**∆Female**	18.86	-18.86	18.03	-85.43	-19.74	-68.47	-92.96	20.93	- excluded
(%-points)	[-20.88,58.60]	[-61.42,23.71]	[-34.81,70.87]	[-213.6,42.77]	[-69.40,29.92]	[-166.9,30.00]	[-223.8,37.89]	[-52.08,93.93]	
**∆Age**		99.16**	116.8***	140.6**	99.77***	128.8*	118.8**	129.5***	158.8***
(years)		**[43.60,154.7]**	**[65.06,168.6]**	**[65.02,216.2]**	**[51.40,148.1]**	**[31.96,225.7]**	**[46.51,191.0]**	**[74.55,184.3]**	**[115.8,201.8]**
**∆Non-institutional accommodation**			-18.91**						-28.74**
(%-points)			**[-31.83,-5.987]**						**[-48.13,-9.342]**
**∆European**				31.24					3.953
(%-points)				[-24.28,86.76]					[-45.05,52.96]
**∆African**					-1.179				9.561
(%-points)					[-51.52,49.17]				[-53.02,72.14]
**∆American**						285.6			434.1
(%-points)						[-1356.7,1927.8]			[-508.2,1376.3]
**∆Asian**							-39.23		
(%-points)							[-107.7,29.24]		
**∆Other**								-91.87	- excluded
(%-points)								[-216.2,32.49]	
Intercept (ß0)	1108.4***	725.7**	535.3**	496.9	721.0**	237.1	367.1	1189.1**	61.64
	**[726.4,1490.4]**	**[298.8,1152.7]**	**[158.7,912.0]**	[-90.20,1083.9]	**[247.8,1194.1]**	[-706.4,1180.6]	[-366.0,1100.3]	**[482.8,1895.4]**	[-557.2,680.5]
					**Model fit**				
F stat. (Model df)	11.04 (2)	25.03 (3)	95.99 (4)	43.32 (4)	32.67 (4)	16.82 (4)	15.66 (4)	49.6 (4)	30.05 (5)
Model sig	**	***	***	***	***	***	***	***	***
Adj. R-squared	58.6	68.2	71.7	74	68.3	49.7	72.8	74.9	55.7
root MSE	194.6	176.2	173.7	166.8	184.1	250	170.9	165.1	242.6
D-W-statistic	1.902	1.878	1.924	1.967	1.879	1.413	1.904	1.926	1.527
N clusters	16	16	16	16	16	17	16	16	17

95% confidence intervals in brackets;

* p<0.05;

** p<0.01;

*** p<0.001; calculated from robust standard errors, adjusted for N clusters. Estimates derived from univariate GLS linear regression models (Prais-Winsten-Regression). Variation inflation factors VIF < 10 in all models. M9: variables excluded due to VIF > 11. D-W-statistic: transformed Durbin-Watson statistic.

### Effect of amendments to the access policy on expenditure differences

As shown by the estimates of the segmented GLS linear regression models, the first policy amendment in 1997 significantly increased the difference in per capita health expenditure between the two groups (*∆IR*_*t*_) compared to the pre-reform period after controlling for secular trends and differences (between exposed and unexposed) in underlying need (age, sex and housing). The immediate increase attributable to the reform ranged between 600.0 [212.6; 986.2] Euros in age-adjusted models and 867.0 [390.9; 1342.5] Euros per capita in sex-adjusted models, corrected for pre-existing secular trends in expenditure differences (Table 4). Despite a decreasing secular trend for the observation period (1994–2013), there was a significant upward trend in the between-group difference in per capita health expenditure in the post-reform period (1997–2013), ranging between 387.0 [186.5; 587.2] and 590.0 [316.7; 862.8] Euros per capita per year depending on the choice of co-variable (Table 4). This trend, but not the immediate policy effect, was attenuated when adjusting for between-group differences in the type of housing facilities. These results remained unaffected by adjustment for between-group differences in age *and* housing facilities in one model (*estimates not shown*).

**Table 4:** Crude and adjusted estimates for effects of restrictive reforms on level and trends in the dependent variable “absolute difference in per capita health expenditure (∆*IR*_*t*_)” in Euros.

	REFORM 1 (1997)				REFORM2 (2007)			
	M1	M2	M3	M4	M1	M2	M3	M4
	Crude	Adj. f. Age	Adj. f. sex	Adj. f. housing	Crude	Adj. f. Age	Adj. f.sex	Adj. f. housing
Secular trend	-536.7***	-583.2***	-441.8***	-404.8	-29.11	-23.05	-30.42	-17.23
	**[-711.5,-361.9]**	**[-850.1,-316.3]**	**[-615.9,-267.6]**	[-915.6,106.1]	[-60.79,2.570]	[-48.84,2.745]	[-69.03,8.188]	[-61.06,26.61]
Change in level of outcome (post- vs. pre-reform period)	862.8**	599.4**	866.7**	854.2**	249.1	-45.44	267.3	182.2
	**[347.5,1378.2]**	**[212.6,986.2]**	**[390.9,1342.5]**	**[334.5,1373.9]**	[-108.6,606.7]	[-380.8,289.9]	[-209.5,744.2]	[-219.2,583.5]
Annual change in trend after reform	496.4***	589.7***	386.9***	351.5	-72.05*	202.6*	-68.28	-102.0*
	**[318.8,674.0]**	**[316.7,862.8]**	**[186.5,587.2]**	[-207.8,910.7]	**[-127.1,-17.03]**	**[7.679,397.5]**	[-138.5,1.928]	**[-184.1,-19.88]**
Age		103.3**				307.3**		
		[42.30,164.3]				**[90.45,524.1]**		
Female			-24.48				6.377	
			[-61.34,12.38]				[-44.72,57.47]	
Non-institutionalised				-10.16				-12.41
				[-46.76,26.44]				[-39.12,14.29]
Interecept (ß0)	1630.9***	1781.5***	1270.8***	1129.5	882.8***	1103.0***	943.5**	507
	**[1316.2,1945.6]**	**[1119.9,2443.0]**	**[741.9,1799.7]**	[-637.8,2896.8]	**[639.2,1126.5]**	**[889.0,1317.1]**	**[369.0,1518.0]**	[-347.1,1361.1]
adjusted R-squared (%)	57.6	61.6	57.3	55.9	55.8	67.7	51.9	54.7
F statistic (Model df)	21.73 (3)	13.45 (4)	57.67 (4)	23.99 (4)	20.26 (3)	41.03 (4)	13.04 (4)	27.68 (4)
Model sig.	***	***	***	***	***	***	***	***
root MSE	203	189	205.5	209.4	186.2	161.7	194.2	188.5
Durbin Watson statistic	1.899	1.841	1.924	1.945	1.885	1.99	1.893	1.882
N clusters	17	17	17	17	16	16	16	16

95% confidence intervals in brackets;

* p<0.05;

** p<0.01;

*** p<0.001;

calculated from robust standard errors, adjusted for N clusters. Estimates derived from segmented GLS linear regression models (Prais-Winsten-Regression). Variation inflation factors VIF < 10 in all models. The pre-reform period is 1994–1996 for REFORM1 and 1994–2006 for REFORM2. The post-reform period is 1997–2013 for REFORM1 and 2007–2013 for REFORM2.

The second amendment (2007) had no significant effect on the outcome. After the reform, there was a significant increase in outcome when adjusting for between-group differences in age in addition to secular trends, and a significant decrease when additionally adjusting for between-group differences in housing.

## Discussion

Our aim was to analyse the effects of restricted access to health care on incident health expenditures on AS&R in Germany (1994–2013) while considering differences in underlying needs between AS&R exposed to restrictions and those who were not (anymore) exposed to such restrictions. We further aimed to evaluate the effect of restrictive policy amendments during the observation period on expenditure differences between the two groups. To the best of our knowledge, this is the first study comparing health expenditures on AS&R with different entitlements in European countries.

We found evidence in support of claims that the cost of exclusion from health care and other welfare services among AS&R is ultimately higher (in terms of incident health expenditures) than granting regular access to needed services. Contrary to lines of arguments in the public discourse, per capita health expenditures in the group exposed to restricted access were higher than in the group with regular access throughout two decades, except for two observations were the opposite was the case. These differences in health expenditures could not completely be explained by differences in need as measured by the variables available in official data. We also found that the first restrictive policy amendment in 1997, which increased the “waiting time” to regular access from 12 to 36 months, significantly increased the level (and partly) trend of expenditure differences between the groups in the post-reform period (i.e. 1997–2014), adjusted for underlying secular trends and need differences between exposed and unexposed. No such effect could be found for the second restrictive policy amendment.

Our study meets several criteria [24] supporting that the observed associations between restricted entitlements and health care expenditures are causal. These comprise the existence of plausible pathways (Fig 2); temporality (the exposure precedes the outcome); a dose-response relationship (the first restrictive policy amendment which prolonged “waiting time” from 12 to 36 months increased the differences in costs between exposed and unexposed, Table 4); consistency over time (the association has been widely consistent over two decades, Fig 6); and consistency in case of crossing-over the exposure (as was the case between 1996 and 2001, when those entitled to regular access in 1996 (Fig 3) were legally “shifted back” to restricted access by the first policy amendment in 1997 without that the direction of the association observed in the pre-reform period changed in the period thereafter (Fig 4)).

Per capita health expenditures 1994–2013 were 40% higher among the restricted access group compared to the expenditures in the group with regular access (1994–2013), and these differences could not be explained *purely* by differences in need. The absolute effect of the restriction for the whole period (1994–2013) amounted to 375.80 Euros per capita and year in absolute terms. Excess expenditures attributable to the restriction were substantial (1.560 billion Euros), and corresponded to 22.2% of total health expenditures in the whole population of AS&R between 1994 and 2013. Assuming a causal relationship as outlined above, these could have been averted over the two decades in the absence of restricted access to health care and other welfare services.

However, differences in entitlements on access to health care can only be seen as a necessary, but not as a sufficient, cause of differences in health care expenditures. A large proportion (50–75%) of the variation in *∆IR*_*t*_ over time could be explained by differences in *∆NEED* variables over time. Differences between exposed and unexposed in the proportion of AS&R from different continents of origin did not significantly contribute to differences in health care expenditures, suggesting that pre- and peri-migration factors do not play a major role in explaining health expenditure differences in the host country.

Except for age, the only need variable that significantly explained differences in health expenditures between those with restricted access and those with regular access was a modifiable post-migration factor: an increase in the proportion of AS&R living non-institutional accommodation in the group with restricted access was associated with lower expenditures in the same group. In other words: increasing the proportion of AS&R located in non-institutional accommodation among the group with restricted access reduced the expenditure differences between the groups. The associations were confirmed when analysing the relationship between changes in *AFe* and *∆NEED* variables over time (S5 Table).

In line with the hypothesised pathways (Fig 2), this suggests that the differences in living conditions that come along with the different entitlements are partially be responsible for differences in health care expenditures, adjusted for the other factors in the model (Table 3). This is a plausible finding in light of epidemiological studies which suggest that institutionalised accommodation is associated with worse health status among AS&R [10], and in light of rigorous qualitative studies which illuminate how stressful AS&R living in institutionalised housing facilities in Germany perceive their housing environment [18]. It also supports previous claims that there is no health benefit in denying health care and other existential rights to AS&R through “othering” processes [19].

### Strengths and Limitations

The main strength of our analysis is that we had access to nationally representative census data on AS&R for a 20-year time period. This allowed us to assess the relationship between entitlements on access to health care and health care expenditures in a quasi-experimental, historically prospective time series design. When analysing the effects of restrictive policy amendments, we could correct for pre-existing secular trends and need differences between exposed an unexposed groups using GLS segmented linear regression. We thus avoided several types of bias that may arise in attempts to analyse policy effects with routine data [25]. In summary, we assessed a contested issue with the best data available.

The main shortcomings of our study are related to the use of aggregate data and the reliance on census data to determine denominators for each group. In absence of the availability of individual data, we needed to rely on aggregate (ecological) data reported by the FSO to test the hypotheses in light of our objectives. This limited the flexibility of the analysis. The fact that we had to rely on census data entails that our denominators are likely to under- or overestimate the actual person-time in each group. Hence, incidence rates were calculated assuming that the denominators remain unchanged throughout a year, which is an unavoidable assumption in absence of individual-level information on entitlements and the exact time of changes thereof. Despite the use of aggregate data, it is worth noting that this study is not prone to the ecological fallacy as long as conclusions on expenditure differences between exposed and unexposed are made on group level. Generalising our findings to individuals within groups would, however, be inappropriate.

The study was also limited by the uncertainty involved in the data used to calculate health expenditures among exposed and unexposed. This uncertainty relates to the fact that the FSO partially aggregates expenditures which are not directly health care related together with health care related ones and reports them in one cost category (Table 1). However, for the group with restricted access this relates only to costs for services granted under section 6 of the Act, which make up only a very small fraction of total health expenditures in this group (S3 Fig). The fact that costs related to treatments in reception centres are not included rather underestimates the expenditures in the group with restricted access. As such, costs for (compulsory) measures performed in the scope of entry screening programmes (e.g. for tuberculosis) can not explain the expenditure differences between exposed and unexposed.

In the group with regular access, the various types of non-health care related costs that are aggregated under services according to Volume 12 of the Social Insurance Code (*Leistungen nach dem 5*.*-9*. *Kapitel SGB XII*) clearly overestimate the true costs for health care in this group, despite the fact that monthly premium-payments are not included (Panel 1). Overall, a possible underestimation of costs in the exposed group (restricted access) and overestimation in the unexposed group (regular access) means that our estimates are conservative.

The pathways (from entitlements to health system outcomes such as costs of care) outlined in the introduction and in Fig 2 are complex, and our analysis could only approximate few of the relevant factors needed to investigate the hypothesised links. In particular, no representative data on “need” in terms of morbidity exists in Germany for AS&R, since this population group is not part of the routine health monitoring [26]. To disentangle the effects of restrictions on access to health care from the effects of entitlements to other welfare services would require detailed prospective, individual-level data on need and other co-variables, which does however not exist on A&R in Germany. The available data does not provide sufficient detail to examine other plausible influences on expenditure differences related to the asylum-seeking process. The stress of length of receiving a decision on the asylum-claim or the desire to receive services before pending deportation could be two potential reasons that service costs were greater in the group with restricted access. However, these factors apply to both groups (exposed and unexposed) because both are equal with respect to their (precarious) legal residence status and the pending decision on their asylum-claim.

In 2005, the German welfare system underwent a major reform. Welfare benefits previously categorised under the Federal Social Security Act (*Bundessozialhilfegesetz*) were subsumed, amended and disaggregated thereafter under services according to Volume 12 of the Social Insurance Code (*Leistungen nach dem 5*.*-9*. *Kapitel SGB XII*). According to the FSO, however, the comparability of expenditure data before and after 2005 is given as long as aggregate expenditure categories (and not specific sub-categories) are compared over time [27], as done in this study.

### Implications for the discourse on access to health care for AS&R

Our study has several implications for the current policy discourse on access to health care for AS&R in Germany and other countries which impose restrictions on access to health care for this population. First, our findings confirm previous claims of CSOs that access restrictions are associated with higher expenditures [22]. Taking the group of AS&R with regular access as comparison group, while adjusting for need differences, there is no evidence that the restrictions in Germany have “saved” public money in the last two decades. On the contrary, our results support claims that the restrictions may have ultimately increased costs e.g. due to delayed care, focus on treatment of acute conditions instead of prevention and health promotion, reliance on expert opinion of public health officials on decisions whether treatments are “medically indicated” in light of the AsylbLG or “dispensable”, and higher administrative costs entailed by the restrictive parallel system with its own funding, purchasing, and re-imbursement schemes.

Second, the ongoing discourse on health care for AS&R in Germany would benefit from taking a more rationale, evidence-informed perspective. On March 1, 2015 a third amendment of the AsylbLG reduced the “waiting time” to regular access from 48 months to 15 months. In light of our findings, which have shown that level and trend of expenditure differences increased in the aftermath of the 1997 amendment, concerns that the *reduction* of “waiting time” to regular access would dramatically increase costs seem to be unjustified. Our approach could be repeated in near future to monitor the effects of this recent reform.

In order to overcome the voucher-based administrative barriers on access to health care (not the legal ones which restrict the depth of coverage with services), the Federal Council of Germany passed an agreement between all federal states in December 2014. It explores the possibility of introducing health insurance cards for AS&R with restricted access, following a model which has been developed in a small federal state in 2005 and has become known as the “Bremer Model”. As a consequence, consultations are ongoing in many German federal states on the pros and cons of introducing cards while upholding the restrictions on the depth of coverage with services. Some federal state ministers argue that eliminating the restrictions and providing the same coverage as for the general population would unavoidably increase health care expenditures for AS&R [28]. Based on our findings there is no evidence for such claims.

Finally, the controversial and partially populist debates on the potential (financial) consequences of granting access to health care for AS&R underline the urgent need for high quality individual-level data on AS&R in Germany for scientific purposes, in order to enable evidence-informed, rationale health policy making for this vulnerable and marginalised part of the population.

## Supporting Information

S1 Appendix Supplementary information on methods. (DOC)

S2 Appendix Regression diagnostics. (DOC)

S1 Fig Incidence rate ratio of health expenditures among asylum-seekers and refugees in Germany 1994–2013 (restricted vs. regular access). The observations in 1997–1999 were excluded from the analysis because the group with regular access (on 31 Dec) was zero.
(TIF)

S2 Fig Annual attributable fraction among the exposed, and among the total population, for per capita health expenditures among asylum-seekers and refugees in Germany (1994–2013). The observations in 1997–1999 were excluded from the analysis because the group with regular access (on 31 Dec) was zero.
(TIF)

S3 Fig Per capita expenditures on asylum-seekers and refugees in Germany with restricted access to health care and fractions of costs according to different sections of the Asylum-Seekers Benefits Act (AsylbLG § 4 vs. §6) during the observation period (1994–2013). Restricted access: refers to access to health care according to sections 4 and 6 of the Asylum-Seekers’ Benefits Act (AsylbLG §§4,6). Total costs (AsylbLG §4+§6) are the sum of annual gross expenditures for services according to section 4 (*Leistungen bei Krankheit*, *Schwangerschaft und Geburt*, *AsylbLG §4)* and section 6 (*sonstige Leistungen*, *AsylbLG §6*) of the Act.
(TIF)

S1 Table Continents of origin of the population of asylum-seekers/refugees in Germany by entitlement (1994–2013). The category, “Other”comprises asylum-seekers with nationalities from Australia and Oceania, stateless asylum-seekers, and asylum-seekers for with unknown nationality.
(DOC)

S2 Table Period means and difference in means of per capita health expenditure and need variables by entitlement (1994–2013). *N = Observations. Period mean: 1994–2013. SD: standard deviation. CI:confidence interval. **p-value of a t-test for paired samples testing the null-hypothesis that Delta(period mean) = 0. Restricted access: refers to the population entitled to health care according to sections 4 and 6 of the Asylum Seekers Benefits Act (AsylbLG §§4,6). Regular access: refers to the population specified under section 2 of the Asylum Seekers Benefits Act (AsylbLG §2) who is entitled to services according to the Federal Social Security Act until 2004 (“*Hilfe in besonderen Lebenslagen”*) or services according to Volume 12 of the Social Insurance Code (“*Leistungen nach dem 5*.*-9*. *Kapitel SGB XII”*) after 2004 analogous to the general population. Delta: refers to the difference in period means of variables between restricted and regular access. The category, “Other”comprises asylum-seekers with nationalities from Australia and Oceania, stateless asylum-seekers, and asylum-seekers for with unknown nationality.
(DOC)

S3 Table Unadjusted (crude) estimates for change in ∆per capita health expenditure (Euro) per year or per one unit increase in *∆NEEDt*. 95% confidence intervals in brackets; * p<0.05; ** p<0.01; *** p<0.001; calculated from robust standard errors, adjusted for N clusters. Estimates derived from univariate GLS linear regression models (Prais-Winsten-Regression). The category, “Other”comprises asylum-seekers with nationalities from Australia and Oceania, stateless asylum-seekers, and asylum-seekers for with unknown nationality.
(DOC)

S4 Table Change in the attributable fraction among the exposed (AFe) per year or per one unit increase in *∆NEEDt* (unadjusted estimates). 95% confidence intervals in brackets; * p<0.05; ** p<0.01; *** p<0.001; calculated from robust standard errors, adjusted for N clusters. Estimates derived from univariate GLS linear regression models (Prais-Winsten-Regression). The category, “Others”comprises asylum-seekers with nationalities from Australia and Oceania, stateless asylum-seekers, and asylum-seekers for with unknown nationality.
(DOC)

S5 Table Change in the attributable fraction among the exposed (AFe) per one unit increase in *∆NEEDt* adjusted for secular trends, age and sex differences. 95% confidence intervals in brackets; * p<0.05; ** p<0.01; *** p<0.001; calculated from robust standard errors, adjusted for N clusters. Estimates derived from univariate GLS linear regression models (Prais-Winsten-Regression). The category, “Others”comprises asylum-seekers with nationalities from Australia and Oceania, stateless asylum-seekers, and asylum-seekers for with unknown nationality.
(DOC)

## References

[1] Norredam M, Mygind A, Krasnik A: Access to health care for asylum seekers in the European Union—a comparative study of country policies. Eur J Public Health 2006, 16: 286–290. 16230318 10.1093/eurpub/cki191

[2] Bosswick W: Development of Asylum Policy in Germany. Journal of Refugee Studies 2000, 13: 43–60.

[3] Liedtke M: National welfare and asylum in Germany. Critical Social Policy 2002, 22: 479–497.

[4] Pross C: Third Class Medicine: Health Care for Refugees in Germany. Health and Human Rights 1998, 3: 40–53. 10343292

[5] Burnett A, Peel M: Asylum seekers and refugees in Britain: Health needs of asylum seekers and refugees. BMJ: British Medical Journal 2001, 322: 544. 11230074 10.1136/bmj.322.7285.544 PMC1119741

[6] Steel Z, Liddell BJ, Bateman-Steel CR, Zwi AB: Global protection and the health impact of migration interception. PLOS medicine 2011, 8: e1001038. 21695084 10.1371/journal.pmed.1001038 PMC3114866

[7] WHO. Everybody's business: Strengthening Health Systems to Improve Health Outcomes: WHO's Framework for Action 2007 Geneva, World Health Organization.

[8] Ingleby D: Ethnicity, Migration and the 'Social Determinants of Health' Agenda. Psychosocial Intervention 2012, 21: 331–341.

[9] Aday LA, Andersen R: A framework for the study of access to medical care. Health Serv Res 1974, 9: 208–220. 4436074 PMC1071804

[10] Porter M, Haslam N: Predisplacement and postdisplacement factors associated with mental health of refugees and internally displaced persons: a meta-analysis. JAMA 2005, 294: 602–612. 16077055 10.1001/jama.294.5.602

[11] Landesamt für Gesundheit und Soziales Berlin. Epidemiologischer Wochenbericht für die Berichtswoche 02/2015 über die im Land Berlin gemäß IfSG erfassten Infektionskrankheiten 2–12. 2015 Berlin, Landesamt für Gesundheit und Soziales Berlin Epi-Info Wochenbericht.

[12] Niedermeier A, Dreweck C. Windpocken: Zu einer Häufung unter somalischen Asylsuchenden in zwei Aufnahmeeinrichtungen in München 48, 479–481. 2010 Berlin, Robert Koch Institut Epidemiologisches Bulletin.

[13] Takla A, Barth A, Siedler A, Stöcker P, Wichmann O et al: Measles outbreak in an asylum-seekers' shelter in Germany: comparison of the implemented with a hypothetical containment strategy. Epidemiology & Infection 2012, 140: 1589–1598. 22313789 10.1017/S0950268811002597

[14] Goosen S, Stronks K, Kunst AE: Frequent relocations between asylum-seeker centres are associated with mental distress in asylum-seeking children: a longitudinal medical record study. International Journal of Epidemiology 2014, 43: 94–104. 24334208 10.1093/ije/dyt233

[15] The Federal Constitutional Court. Provisions governing basic cash benefits provided for in the Asylum Seekers Benefits Act held unconstitutional. Press release No. 56/2012. 18-7-2012. 1/3/15.

[16] Classen G. Das Asylbewerberleistungsgesetz und das Grundrecht auf ein menschenwürdiges Existenzminimum: Stellungnahme zur Anhörung am 07.02.2011 im Ausschuss für Arbeit und Soziales des Deutschen Bundestages 2011 Berlin, Fluechtlingsrat Berlin e.V.; Förderverein PRO ASYL e.V.

[17] Hocking DC, Kennedy GA, Sundram S: Mental Disorders in Asylum Seekers: The Role of the Refugee Determination Process and Employment. The Journal of nervous and mental disease 2015, 203: 28–32. 25503784 10.1097/NMD.0000000000000230

[18] Behrensen B, Groß V. Auf dem Weg in ein *"normales Leben"*? Eine Analyse der gesundheitlichen Situation von Asylsuchenden in der Region Osnabrück 2004 Osnabrück, University Osnabrück. 2/5/15.

[19] Grove NJ, Zwi AB: Our health and theirs: Forced migration, othering, and public health. Social Science & Medicine 2006, 62: 1931–1942. 16242227 10.1016/j.socscimed.2005.08.061

[20] Schunck R, Reiss K, Razum O: Pathways between perceived discrimination and health among immigrants: evidence from a large national panel survey in Germany. *Ethnicity & health* 2014, 1–18. 10.1080/13557858.2014.932756 24992379

[21] Krieger N, Davey Smith G: "Bodies Count" and Body Counts: Social Epidemiology and Embodying Inequality. Epidemiologic Reviews 2004, 26: 92–103. 15234950 10.1093/epirev/mxh009

[22] Classen G. Stellungnahme zum Gesetzentwurf der Bundesregierung "Entwurf eines Gesetzes zur Änderung des Asylbewerberleistungsgesetzes und des Sozialgerichtgesetzes"—BT-Drs.18/2592 vom 22.09.2014. 50–51 2014 Berlin, Fluechtlingsrat Berlin e.V. 2/3/15.

[23] Zimmerman C, Kiss L, Hossain M: Migration and health: a framework for 21st century policy-making. PLOS medicine 2011, 8: e1001034. 21629681 10.1371/journal.pmed.1001034 PMC3101201

[24] Rothman K, Greenland S: Causation and causal inference In Modern Epidemiology. Edited by Rothman K, Greenland S. Philadelphia: Lippincott-Raven Publishers; 1998:7–28.

[25] Lagarde M: How to do (or not to do)… Assessing the impact of a policy change with routine longitudinal data. Health Policy Plan 2012, 27: 76–83. 21278077 10.1093/heapol/czr004

[26] Schneider C, Mohsenpour A, Joos S, Bozorgmehr K: Health status of and health-care provision to asylum seekers in Germany: protocol for a systematic review and evidence mapping of empirical studies. Syst Rev 2014, 3: 139. 25433520 10.1186/2046-4053-3-139 PMC4259011

[27] Federal Statistics Office. Statistik der Sozialhilfe—Ausgaben und Einnahmen—Methodik [generell]. Available: https://www.gbe-bund.de/gbe10/abrechnung.prc_abr_test_logon?p_uid=gast&p_aid=0&p_knoten=FID&p_sprache=D&p_suchstring=8252::Leistungen%20nach%20dem%205.%20bis%209.%20Kapitel%20SGB%20XII Accessed 08 February 2015.

[28] Landtag Baden-Württemberg. Drucksache 15/4595—Antrag der Abg. Florian Wahl u.a. SPD und Stellungnahme des Ministeriums für Integration: Gesundheitsversorgung von Flüchtlingen in Baden-Württemberg. 15-1-2014.

