# Supplementary material for: Effect of Restricting Access to Health Care on Health Expenditures among Asylum-Seekers and Refugees: A Quasi-Experimental Study in Germany, 1994–2013
Source: PLoS One. 2015 Jul 22;10(7):e0131483. doi: 10.1371/journal.pone.0131483 (PMC4511805; doi:10.1371/journal.pone.0131483)
Supplement: S1 Appendix — (DOC) [file pone.0131483.s001.doc]

**S1 Appendix – Supplementary notes to methods**

**Effect of restricting access to health care on health expenditures among asylum-seekers and refugees: a quasi-experimental study in Germany, 1994-2013**

[Supplementary notes to Methods 2](#__RefHeading___Toc419895889)

[Types of expenditure used to calculate health expenditures on asylum-seekers and refugees in Germany 2](#__RefHeading___Toc419895890)

[Health expenditures for the restricted access group (exposed) 2](#__RefHeading___Toc419895891)

[Health expenditures for the group with regular access (unexposed) 2](#__RefHeading___Toc419895892)

[Health expenditures for the total population 3](#__RefHeading___Toc419895893)

[Measurements and statistical analysis 3](#__RefHeading___Toc419895894)

[Calculation of attributable fractions 3](#__RefHeading___Toc419895895)

**Address for correspondence**

Dr. med. Kayvan Bozorgmehr (MD, MSc)

University Hospital Heidelberg, Voßstr.2, Geb. 37, 69115 Heidelberg

Phone: +49 6221 56 38581, Email: [kayvan.bozorgmehr@med.uni-heidelberg.de](mailto:kayvan.bozorgmehr@med.uni-heidelberg.de)

# Supplementary notes to Methods

## Types of expenditure used to calculate health expenditures on asylum-seekers and refugees in Germany

We used different types of expenditure data, provided by the Federal Statistics Office since 1994, to calculate health expenditures among exposed and unexposed AS&R respectively.

### Health expenditures for the restricted access group (exposed)

Health expenditures for AS&R with restricted access were calculated as the sum of annual gross expenditures for services according to section 4 (*Leistungen bei Krankheit, Schwangerschaft und Geb*ur*t, AsylbLG §4*) and section 6 (*sonstige Leistungen, AsylbLG §6*) of the Asylum-Seekers’ Benefits Act. Expenditures under section 4 consist of costs for in-patient and out-patient treatment of acute or painful conditions (including dental care), vaccination, and preventive maternal care services including costs of delivery. Treatment costs of other conditions are categorised under section 6 and may also include costs for medical aids, or nursing support, but these constitute only small fractions of the total costs (Error: Reference source not found). Not included are health care costs in reception centres (*Landeserstaufnahmestellen*) during the first 6-12 weeks after submission of the asylum application.

### Health expenditures for the group with regular access (unexposed)

Health expenditures for AS&R with regular access were defined as gross annual expenditures for services according to the Federal Social Security Act until 2004 (*Hilfe in besonderen Lebenslagen*), and services according to Volume 12 of the Social Insurance Code (*Leistungen nach dem 5.-9. Kapitel SGB XII*) after 2004. These expenditures consist of the costs for the treatment of all conditions throughout the year (in-patient and out-patient care), i.e. for all the services for which the Statutory Sickness Funds were re-imbursed by the Welfare Agencies. Not included are the monthly contributions to the Statutory Sickness Funds, i.e. the costs exclusively include expenditures for treatment. Estimates are nevertheless conservative in that they rather overestimate the true costs due to aggregation of several services not related to health care.

### Health expenditures for the total population

Were calculated as the sum of all health expenditures on AS&R with restricted and regular access.

Estimates are conservative in that they rather overestimate the true costs due to aggregation of several services not related to health care. These are particularly costs categorised under “AsylbLG §6” and “Hilfe in besonderen Lebenslagen”/ “Leistungen nach dem 5.-9. Kapitel SGB XII”, which may include other costs such as long-term care, medical aids, disability related costs of integration etc. Not included are health care costs in reception centres (*Landeserstaufnahmestellen*) during the first 6-12 weeks after arrival in the country.

## Measurements and statistical analysis

### Calculation of attributable fractions

The attributable fractions among the exposed at measurement occasion t were calculated as *AFe_t = (Equation S.1)_*

where *IR_t_* is the incidence rate at measurement occasion *t* for each group (exposed and unexposed).

The attributable fraction among the total population at measurement occasion t was calculated as

*AFp_t_ = $\frac{\text{IR}_{\text{t, total pop}}\text{. - IR}_{\text{t, unexposed}}}{\text{IR}_{\text{t, total pop.}}}$ _(Equation S.2)_*

where *IR_t, totalpop._* is the incidence rate at measurement occasion *t* for the total population (i.e. the joint *IR_t_* for both exposed and unexposed taken together).
