# Supplementary material for: Effect of Restricting Access to Health Care on Health Expenditures among Asylum-Seekers and Refugees: A Quasi-Experimental Study in Germany, 1994–2013
Source: PLoS One. 2015 Jul 22;10(7):e0131483. doi: 10.1371/journal.pone.0131483 (PMC4511805; doi:10.1371/journal.pone.0131483)
Supplement: S2 Appendix — (DOC) [file pone.0131483.s002.doc]

## S2 Appendix: Regression diagnostics

### Linearity assumption and outlier analysis

Figure S4: Scatter plot and linear fit of percentage-point differences (between exposed and unexposed) in the population of asylum-seekers with EUROPEAN nationality and differences in per capita expenditures on health (between exposed and unexposed)


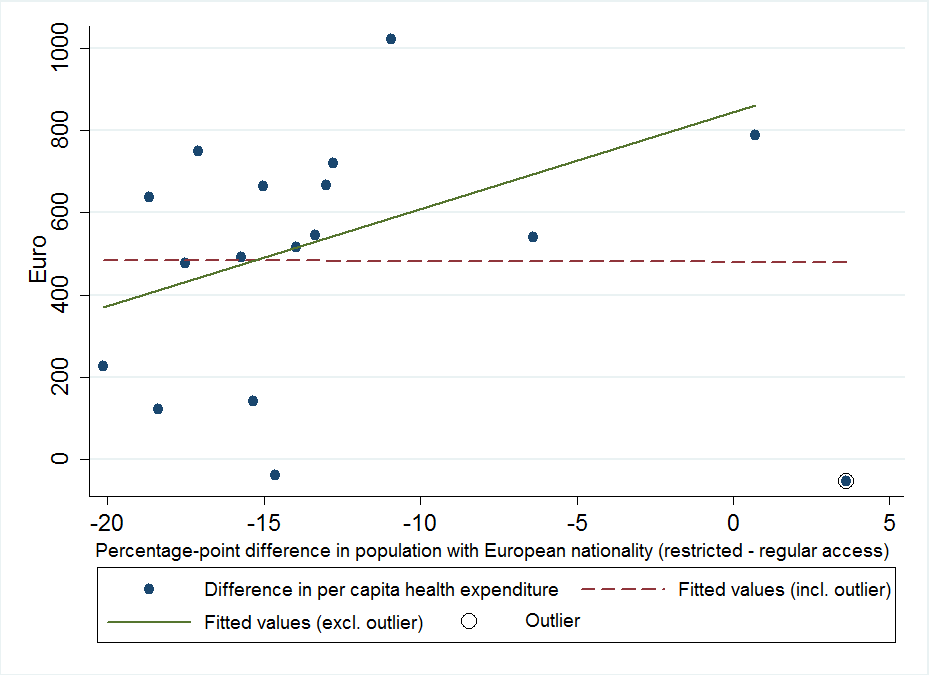


Figure S5: Scatter plot and linear fit of percentage-point differences (between exposed and unexposed) in the population of asylum-seekers with ASIAN nationality and differences in per capita expenditures on health (between exposed and unexposed)


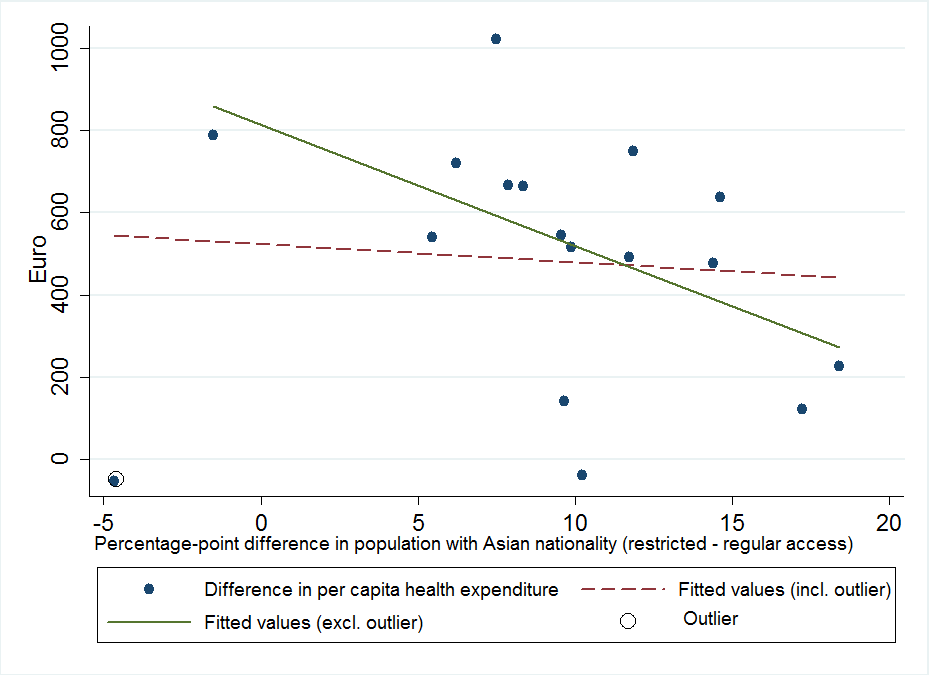


Figure S6: Scatter plot and linear fit of percentage-point differences (between exposed and unexposed) in the population of asylum-seekers with nationalities from the AMERICAN continents and differences in per capita expenditures on health (between exposed and unexposed)


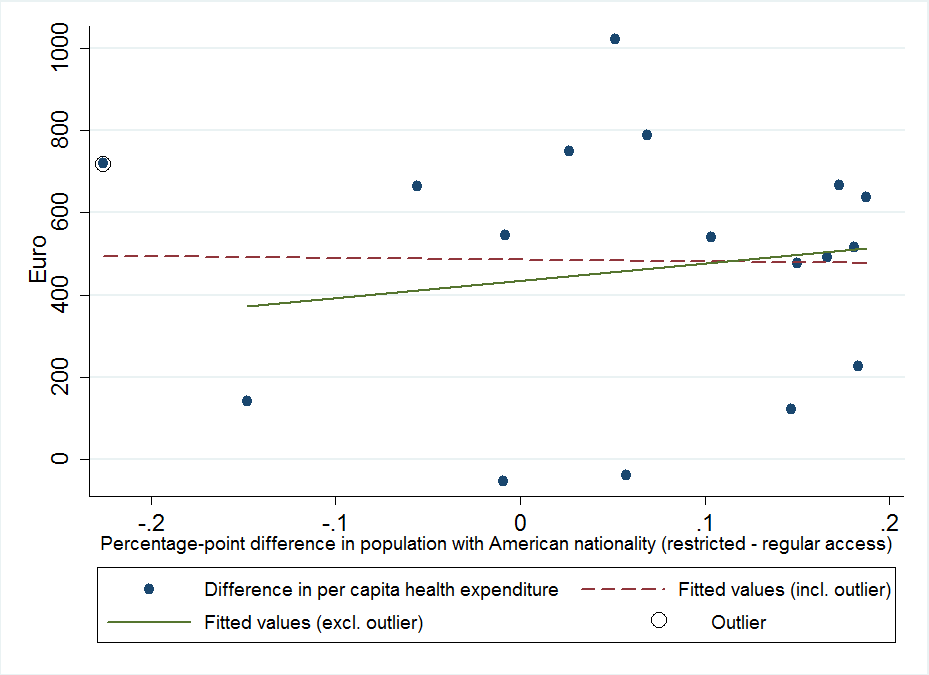


Figure S7: Scatter plot and linear fit of percentage-point differences (between exposed and unexposed) in the population of asylum-seekers with AFRICAN nationality and differences in per capita expenditures on health (between exposed and unexposed)


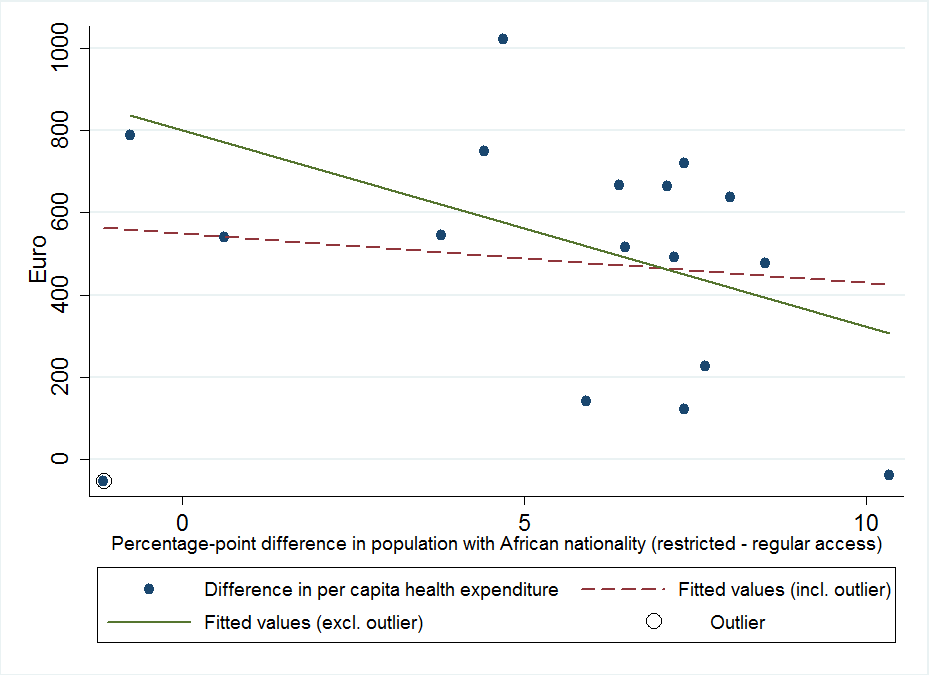


Figure S8: Scatter plot and linear fit of percentage-point differences (between exposed and unexposed) in the population of asylum-seekers with OTHER/UNKNOWN nationalities and differences in per capita expenditures on health (between exposed and unexposed)


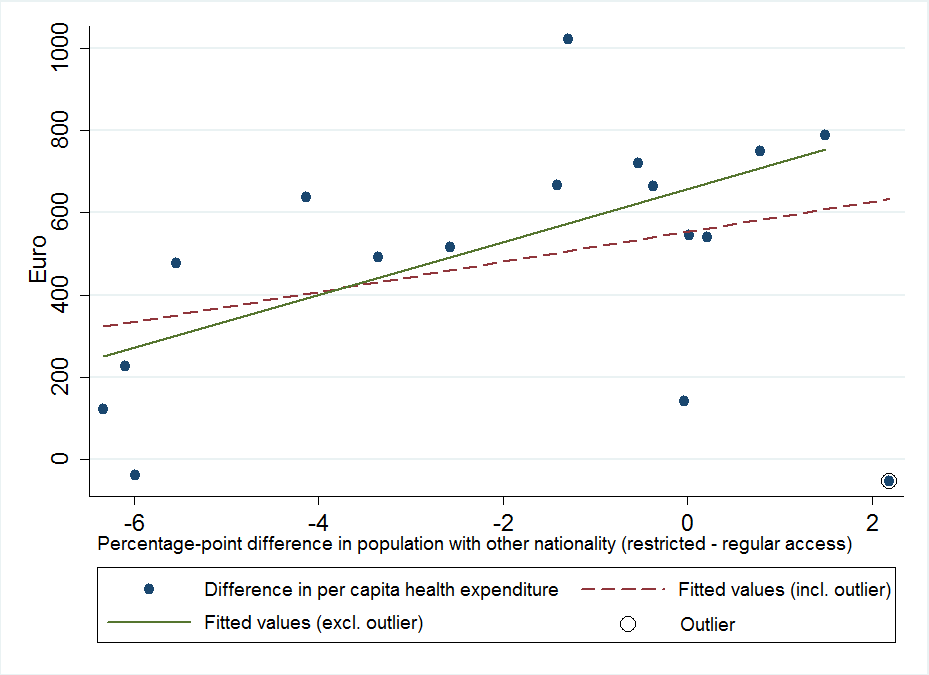


Figure S9: Scatter plot and linear fit of AGE differences in years (between exposed and unexposed) and differences in per capita expenditures on health (between exposed and unexposed)


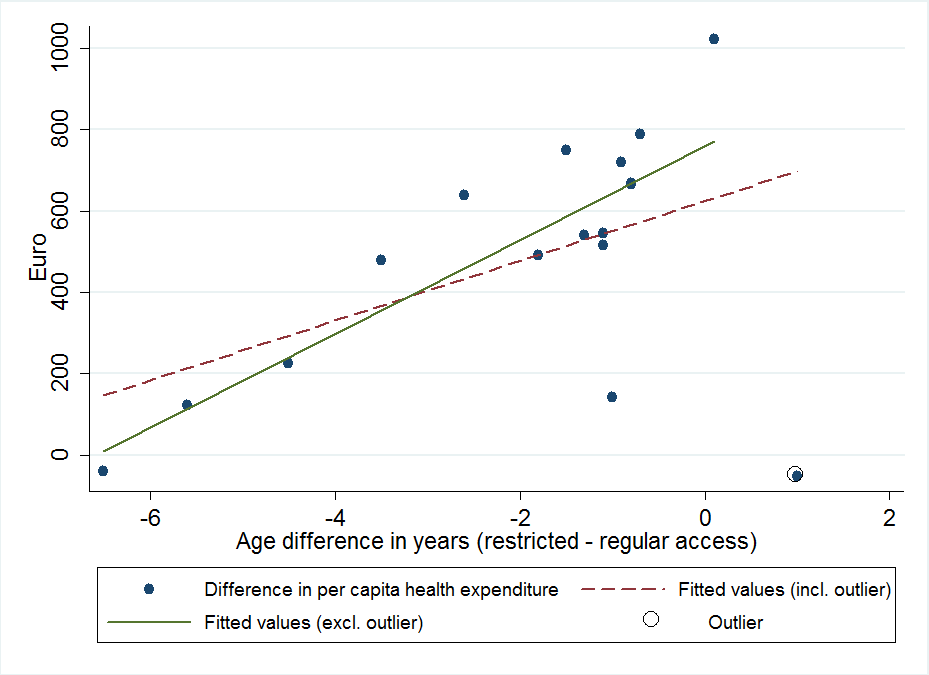


Figure S10: Scatter plot and linear fit of percentage-point differences (between exposed and unexposed) in the proportion of FEMALE asylum-seekers and differences in per capita expenditures on health (between exposed and unexposed)


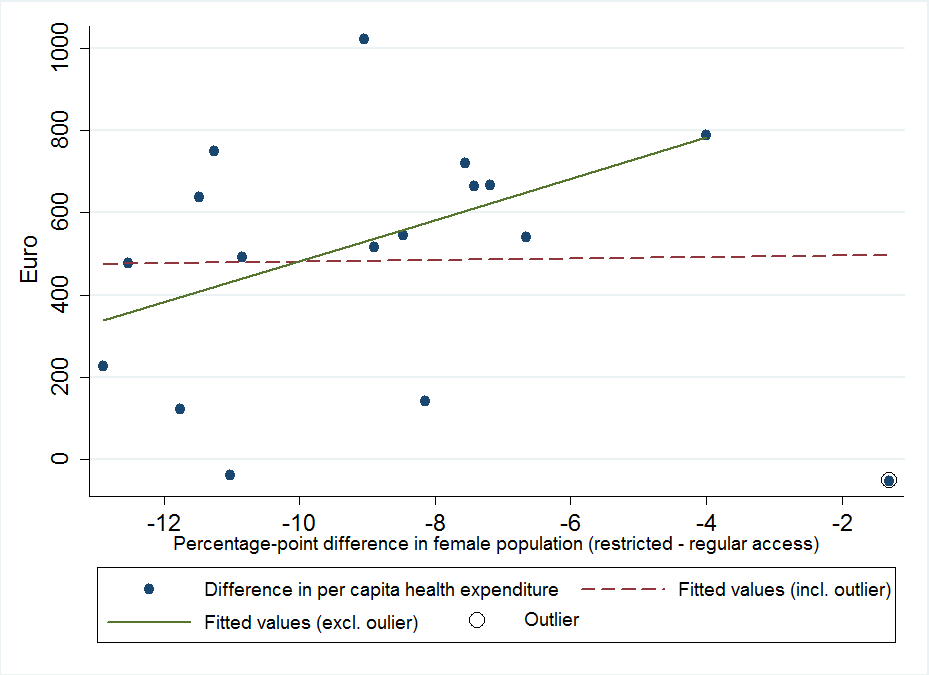


Figure S11: Scatter plot and linear fit of percentage-point differences (between exposed and unexposed) in the proportion of asylum-seekers RESIDING IN NON-INSTITUTIONAL ACCOMMODATION and differences in per capita expenditures on health (between exposed and unexposed)


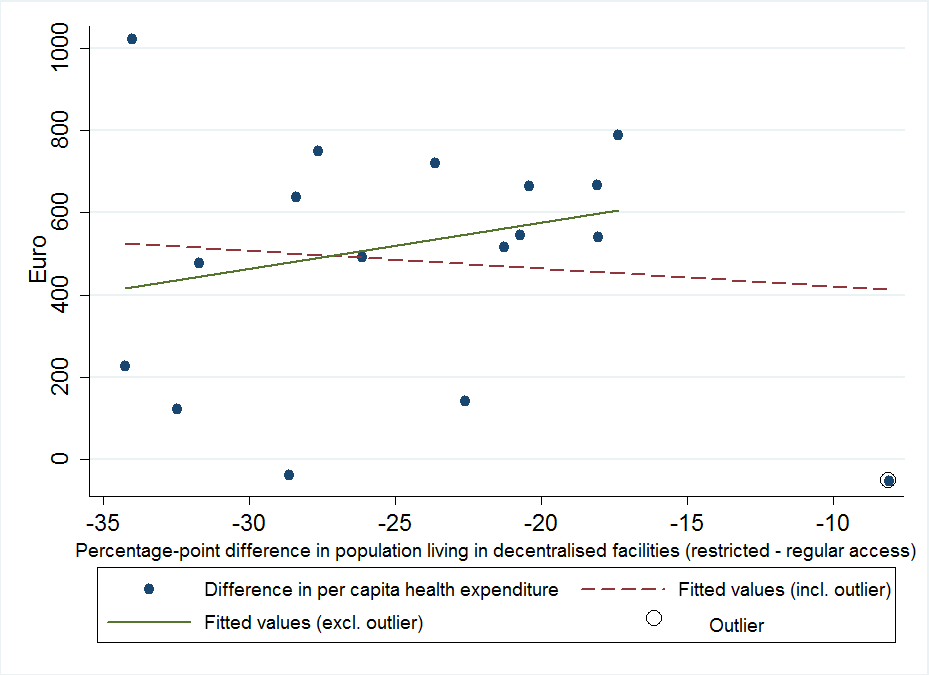


Figure S12: Correlograph analysing stationarity of differences in per capita expenditures (between exposed and unexposed) over time


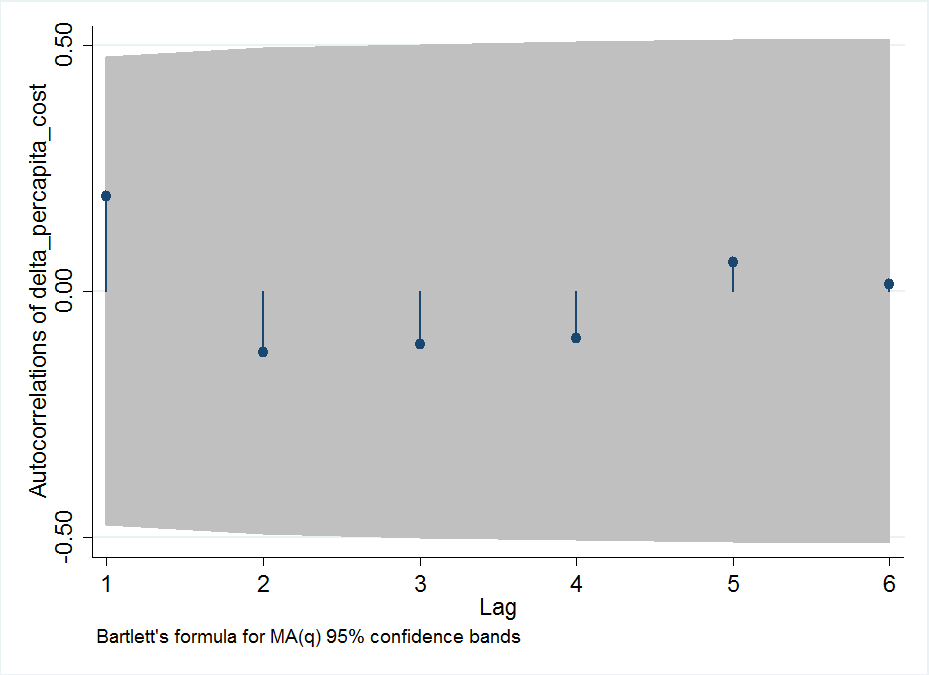


Delta_per_capita_cost= differences in per capita expenditures (between exposed and unexposed). The correlograph indicates that the data is non-stationary. Shaded area constitute 95%confidence intervals.
