# Supplementary figures and images for: Effect of Restricting Access to Health Care on Health Expenditures among Asylum-Seekers and Refugees: A Quasi-Experimental Study in Germany, 1994–2013
Source: PLoS One. 2015 Jul 22;10(7):e0131483. doi: 10.1371/journal.pone.0131483 (PMC4511805; doi:10.1371/journal.pone.0131483)

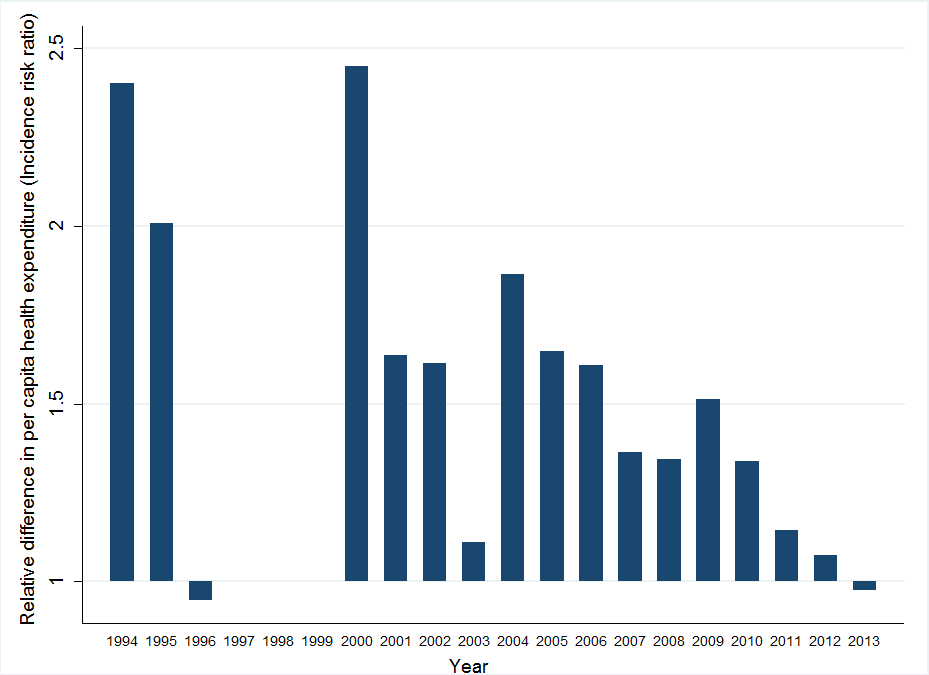

Supplement: S1 Fig — The observations in 1997–1999 were excluded from the analysis because the group with regular access (on 31 Dec) was zero. (TIF) [file pone.0131483.s003.tif]

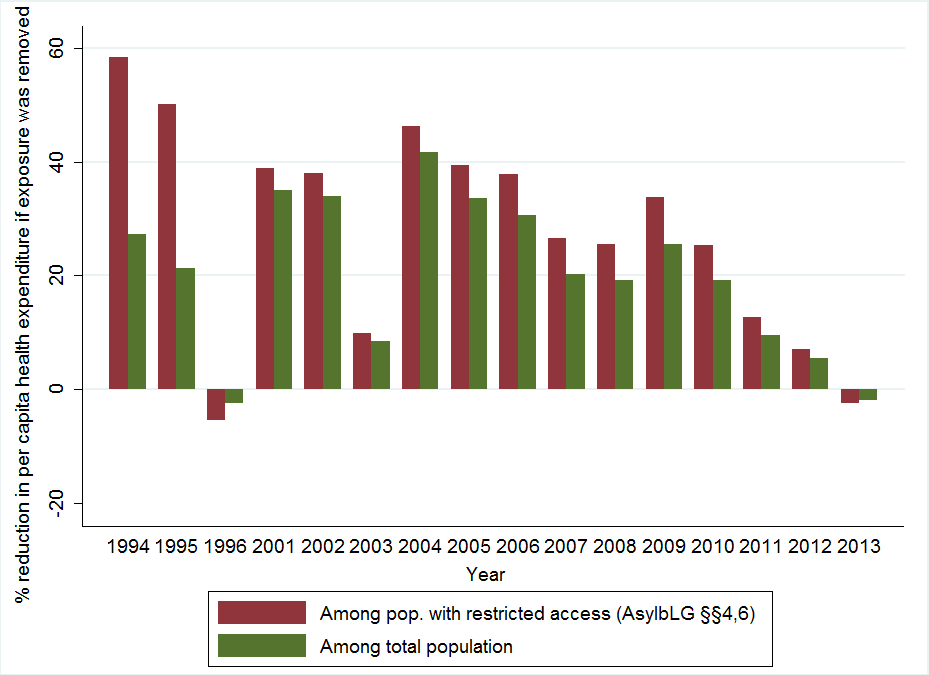

Supplement: S2 Fig — The observations in 1997–1999 were excluded from the analysis because the group with regular access (on 31 Dec) was zero. (TIF) [file pone.0131483.s004.tif]

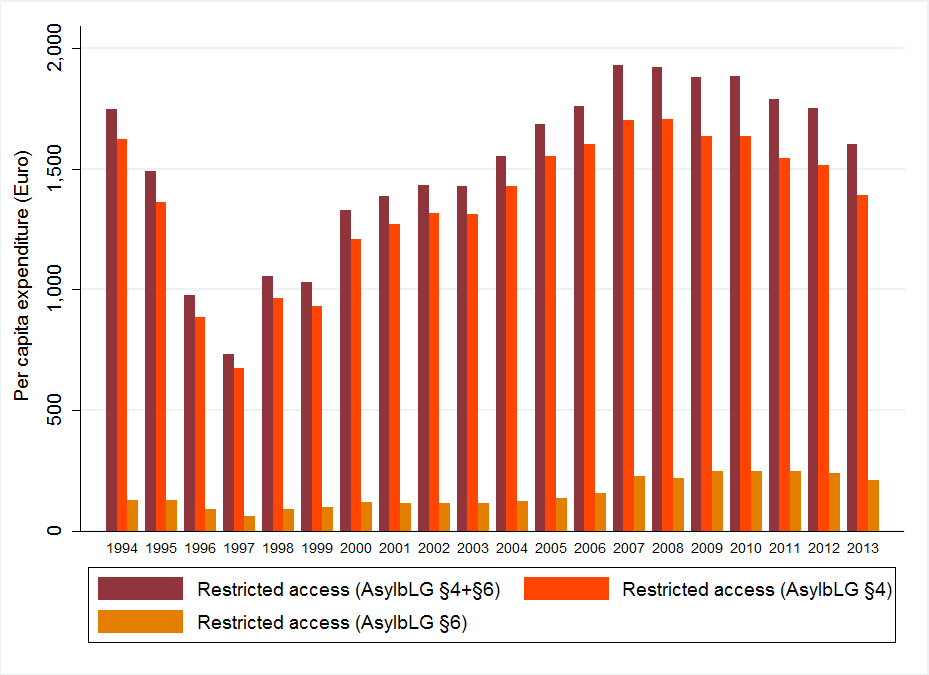

Supplement: S3 Fig — Restricted access: refers to access to health care according to sections 4 and 6 of the Asylum-Seekers’ Benefits Act (AsylbLG §§4,6). Total costs (AsylbLG §4+§6) are the sum of annual gross expenditures for services according to section 4 (Leistungen bei Krankheit, Schwangerschaft und Geburt, AsylbLG §4) and section 6 (sonstige Leistungen, AsylbLG §6) of the Act. (TIF) [file pone.0131483.s005.tif]
