# Supplementary material for: Effect of Restricting Access to Health Care on Health Expenditures among Asylum-Seekers and Refugees: A Quasi-Experimental Study in Germany, 1994–2013
Source: PLoS One. 2015 Jul 22;10(7):e0131483. doi: 10.1371/journal.pone.0131483 (PMC4511805; doi:10.1371/journal.pone.0131483)
Supplement: S1 Table — The category, “Other”comprises asylum-seekers with nationalities from Australia and Oceania, stateless asylum-seekers, and asylum-seekers for with unknown nationality. (DOC) [file pone.0131483.s006.doc]

Table S1: Continents of origin of the population of asylum-seekers/refugees in Germany by entitlement (1994-2013)

|  | **Restricted access** | | | | | | **Regular access** | | | | | |
| --- | --- | --- | --- | --- | --- | --- | --- | --- | --- | --- | --- | --- |
|  |  | **Nationalities - n (%)** | | | | |  | **Nationalities - n (%)** | | | | |
| **Year** | **N** | **Europe** | **Africa** | **America** | **Asia** | **Other** | **N** | **Europe** | **Africa** | **America** | **Asia** | **Other** |
| 1994 | 117,429 | 67,303 (57.3) | 13,527 (11.5) | 163 (0.1) | 30,628 (26.1) | 5,808 (4.9) | 321,189 | 219,207 (68.2) | 21,949 (6.8) | 280 (0.1) | 59,734 (18.6) | 20,019 (6.2) |
| 1995 | 131,820 | 70,111 (53.2) | 13,975 (10.6) | 126 (0.1) | 39,256 (29.8) | 8,352 (6.3) | 357,154 | 250,960 (70.3) | 22,105 (6.2) | 247 (0.1) | 64,047 (17.9) | 19,795 (5.5) |
| 1996 | 226,580 | 147,533 (65.1) | 14,778 (6.5) | 193 (0.1) | 41,430 (18.3) | 22,646 (10) | 263,162 | 161,815 (61.5) | 20,205 (7.7) | 248 (0.1) | 60,366 (22.9) | 20,528 (7.8) |
| 1997 | 486,643 | 299,942 (61.6) | 39,910 (8.2) | 548 (0.1) | 110,159 (22.6) | 36,684 (7.5) | 0 | 0 (0) | 0 () | 0 () | 0 () | 0 () |
| 1998 | 438,873 | 265,939 (60.6) | 35,951 (8.2) | 554 (0.1) | 104,538 (23.8) | 31,891 (7.3) | 0 | 0 (0) | 0 () | 0 () | 0 () | 0 () |
| 1999 | 435,930 | 276364 (63.4) | 35,447 (8.1) | 633 (0.1) | 108,806 (25) | 14,680 (3.4) | 0 | 0 (0) | 0 () | 0 () | 0 () | 0 () |
| 2000 | 318,238 | 182,898 (57.5) | 27,077 (8.5) | 610 (0.2) | 94,686 (29.8) | 12,967 (4.1) | 33,404 | 18,959 (56.8) | 3,097 (9.3) | 41 (0.1) | 10,444 (31.3) | 863 (2.6) |
| 2001 | 266,064 | 139,353 (52.4) | 25,289 (9.5) | 585 (0.2) | 90,959 (34.2) | 9,878 (3.7) | 48,052 | 28,237 (58.8) | 4,276 (8.9) | 56 (0.1) | 13,803 (28.7) | 1,680 (3.5) |
| 2002 | 233,143 | 110,280 (47.3) | 26,097 (11.2) | 504 (0.2) | 86,824 (37.2) | 9,438 (4) | 45,449 | 27,566 (60.7) | 3,370 (7.4) | 102 (0.2) | 12,583 (27.7) | 1,828 (4) |
| 2003 | 222,162 | 98,283 (44.2) | 27,797 (12.5) | 363 (0.2) | 86,110 (38.8) | 9,667 (4.4) | 42,078 | 25,072 (59.6) | 2,780 (6.6) | 131 (0.3) | 12,252 (29.1) | 1,846 (4.4) |
| 2004 | 190,535 | 80,528 (42.3) | 25,877 (13.6) | 335 (0.2) | 75,296 (39.5) | 8,499 (4.5) | 39,613 | 21,807 (55.1) | 2,474 (6.2) | 159 (0.4) | 13,197 (33.3) | 1,976 (5) |
| 2005 | 165,016 | 66,448 (40.3) | 22,740 (13.8) | 287 (0.2) | 65,904 (39.9) | 9,637 (5.8) | 46,106 | 25,484 (55.3) | 3,089 (6.7) | 106 (0.2) | 14,563 (31.6) | 2,864 (6.2) |
| 2006 | 140,650 | 55,891 (39.7) | 18,737 (13.3) | 501 (0.4) | 56,701 (40.3) | 8,820 (6.3) | 52,912 | 27,901 (52.7) | 3,675 (6.9) | 97 (0.2) | 17,174 (32.5) | 4,065 (7.7) |
| 2007 | 107,200 | 38,825 (36.2) | 14,364 (13.4) | 324 (0.3) | 45,825 (42.7) | 7,862 (7.3) | 46,100 | 23,127 (50.2) | 3,191 (6.9) | 56 (0.1) | 15,159 (32.9) | 4,567 (9.9) |
| 2008 | 87,894 | 27,488 (31.3) | 12,579 (14.3) | 289 (0.3) | 40,387 (45.9) | 7,151 (8.1) | 39,971 | 18,784 (47) | 2,846 (7.1) | 65 (0.2) | 13,683 (34.2) | 4,593 (11.5) |
| 2009 | 81,314 | 23,056 (28.4) | 12,369 (15.2) | 295 (0.4) | 39,523 (48.6) | 6,071 (7.5) | 39,921 | 18,773 (47) | 2,879 (7.2) | 70 (0.2) | 13,570 (34) | 4,629 (11.6) |
| 2010 | 90,546 | 27,002 (29.8) | 13,863 (15.3) | 277 (0.3) | 43,367 (47.9) | 6,037 (6.7) | 39,751 | 18,812 (47.3) | 2,700 (6.8) | 62 (0.2) | 13,325 (33.5) | 4,852 (12.2) |
| 2011 | 103,767 | 28,812 (27.8) | 15,108 (14.6) | 351 (0.3) | 53,505 (51.6) | 5,991 (5.8) | 39,920 | 19,115 (47.9) | 2,760 (6.9) | 62 (0.2) | 13,247 (33.2) | 4,736 (11.9) |
| 2012 | 128,284 | 41,090 (32) | 18,487 (14.4) | 407 (0.3) | 62,898 (49) | 5,402 (4.2) | 36,960 | 18,628 (50.4) | 2,617 (7.1) | 63 (0.2) | 11,757 (31.8) | 3,895 (10.5) |
| 2013 | 188,624 | 67,254 (35.7) | 34,601 (18.3) | 487 (0.3) | 79,966 (42.4) | 6,316 (3.3) | 36,369 | 18,286 (50.3) | 2,912 (8) | 73 (0.2) | 11,705 (32.2) | 3,393 (9.3) |
|  |  |  |  |  |  |  |  |  |  |  |  |  |

The category „Other“ comprises asylum-seekers with nationalities from Australia and Oceania, stateless asylum-seekers, and asylum-seekers for with unknown nationality.
