# Supplementary material for: Effect of Restricting Access to Health Care on Health Expenditures among Asylum-Seekers and Refugees: A Quasi-Experimental Study in Germany, 1994–2013
Source: PLoS One. 2015 Jul 22;10(7):e0131483. doi: 10.1371/journal.pone.0131483 (PMC4511805; doi:10.1371/journal.pone.0131483)
Supplement: S2 Table — *N = Observations. Period mean: 1994–2013. SD: standard deviation. CI:confidence interval. **p-value of a t-test for paired samples testing the null-hypothesis that Delta(period mean) = 0. Restricted access: refers to the population entitled to health care according to sections 4 and 6 of the Asylum Seekers Benefits Act (AsylbLG §§4,6). Regular access: refers to the population specified under section 2 of the Asylum Seekers Benefits Act (AsylbLG §2) who is entitled to services according to the Federal Social Security Act until 2004 (“Hilfe in besonderen Lebenslagen”) or services according to Volume 12 of the Social Insurance Code (“Leistungen nach dem 5.-9. Kapitel SGB XII”) after 2004 analogous to the general population. Delta: refers to the difference in period means of variables between restricted and regular access. The category, “Other”comprises asylum-seekers with nationalities from Australia and Oceania, stateless asylum-seekers, and asylum-seekers for with unknown nationality. (DOC) [file pone.0131483.s007.doc]

Table S2: Period means and difference in means of per capita health expenditure and need variables by entitlement (1994-2013)

|  | Restricted access | | | | Regular access | | | | Delta (restricted - regular access) | | | | |
| --- | --- | --- | --- | --- | --- | --- | --- | --- | --- | --- | --- | --- | --- |
| Variable | N* | Period mean | *SD* | 95%CI | N* | Period mean | *SD* | 95%CI | Period mean | *SE* | 95%CI | t-statistic | p-value** |
| Per capita health expenditure (Euro) | 20 | 1518.9 | *348.7* | [1355.7 ; 1682.1] | 19 | 1018.3 | *483.8* | [785.1 ; 1251.5] | 500.6 | *134.5* | [228.1 ; 773.2] | 3.722 | 0.0007 |
| Female (%) | 20 | 38.7 | *2.3* | [37.6 ; 39.8] | 17 | 47.1 | *2.3* | [46 ; 48.3] | -8.5 | *0.8* | [-10 ; -6.9] | -11.187 | <0.0001 |
| Age (years) | 20 | 24.7 | *1.2* | [24.1 ; 25.3] | 17 | 26.9 | *2.8* | [25.4 ; 28.3] | -2.2 | *0.7* | [-3.6 ; -0.8] | -3.135 | 0.0035 |
| Non-institutionalised housing (%) | 20 | 50 | *5.9* | [47.2 ; 52.7] | 17 | 72.8 | *4.2* | [70.7 ; 75] | -22.9 | *1.7* | [-26.3 ; -19.4] | -13.317 | <0.0001 |
| *Nationality* |  |  |  |  |  |  |  |  |  |  |  |  |  |
| European (%) | 20 | 45.3 | *12.7* | [39.3 ; 51.3] | 17 | 55.2 | *7.2* | [51.5 ; 58.9] | -9.9 | *3.5* | [-17 ; -2.9] | -2.848 | 0.0073 |
| African (%) | 20 | 12.1 | *3.1* | [10.6 ; 13.5] | 17 | 7.2 | *0.8* | [6.8 ; 7.7] | 4.8 | *0.8* | [3.2 ; 6.4] | 6.202 | <0.0001 |
| American (%) | 20 | 0.2 | *0.1* | [0.2 ; 0.3] | 17 | 0.2 | *0.1* | [0.1 ; 0.2] | 0.045 | *0.030* | [-0.01 ; 0.1] | 1.548 | 0.1307 |
| Asian (%) | 20 | 36.7 | *10* | [32 ; 41.3] | 17 | 29.7 | *5.2* | [27.1 ; 32.4] | 6.9 | *2.7* | [1.5 ; 12.4] | 2.589 | 0.0139 |
| Other (%) | 20 | 5.8 | *1.8* | [4.9 ; 6.6] | 17 | 7.6 | *3.2* | [6 ; 9.3] | -1.9 | *0.8* | [-3.6 ; -0.2] | -2.219 | 0.0331 |
|  |  |  |  |  |  |  |  |  |  |  |  |  |  |

*N=Observations. Period mean: 1994-2013. SD: standard deviation. CI:confidence interval. **p-value of a t-test for paired samples testing the null-hypothesis that Delta(period mean)=0. **Restricted access:** refers to the population entitled to health care according to sections 4 and 6 of the Asylum Seekers Benefits Act (AsylbLG §§4,6). **Regular access:** refers to the population specified under section 2 of the Asylum Seekers Benefits Act (AsylbLG §2) who is entitled to services according to the Federal Social Security Act until 2004 (“*Hilfe in besonderen Lebenslagen”*) or services according to Volume 12 of the Social Insurance Code (“*Leistungen nach dem 5.-9. Kapitel SGB XII”*) after 2004 analogous to the general population. Delta: refers to the difference in period means of variables between restricted and regular access. The category „Other“ comprises asylum-seekers with nationalities from Australia and Oceania, stateless asylum-seekers, and asylum-seekers for with unknown nationality.
