# Supplementary material for: Effect of Restricting Access to Health Care on Health Expenditures among Asylum-Seekers and Refugees: A Quasi-Experimental Study in Germany, 1994–2013
Source: PLoS One. 2015 Jul 22;10(7):e0131483. doi: 10.1371/journal.pone.0131483 (PMC4511805; doi:10.1371/journal.pone.0131483)
Supplement: S3 Table — 95% confidence intervals in brackets; * p<0.05; ** p<0.01; *** p<0.001; calculated from robust standard errors, adjusted for N clusters. Estimates derived from univariate GLS linear regression models (Prais-Winsten-Regression). The category, “Other”comprises asylum-seekers with nationalities from Australia and Oceania, stateless asylum-seekers, and asylum-seekers for with unknown nationality. (DOC) [file pone.0131483.s008.doc]

Table S3: Unadjusted (crude) estimates for change in ∆per capita health expenditure (Euro) per year or per one unit increase in ∆*NEEDt*

|  |  | **∆Need variables** | | | | | | | |
| --- | --- | --- | --- | --- | --- | --- | --- | --- | --- |
|  | **Time** | **∆Age** | **∆Female** | **∆Decentralised** | **∆Europe** | **∆Africa** | **∆America** | **∆Asia** | **∆Other** |
|  | (years) | (years) | (percentage-points) | | | | | | |
| Change in ∆per capita health expenditure (in Euro) per one unit increase in time/∆need | **-38.36***** | **120.2***** | **56.46**** | 2.635 | **24.79*** | **-48.79*** | 778.3 | **-27.58*** | **65.87**** |
|  | **[-55.64 ; -21.08]** | **[79.80 ; 160.6]** | **[17.89 ; 95.02]** | [-34.76 ; 40.03] | **[4.925 ; 44.66]** | **[-93.70 ; -3.871]** | [-632.9 ; 2189.4] | **[-52.15 ; -3.017]** | **[21.45 ; 110.3]** |
| Intercept *(ß0)* | **971.8***** | **775.4***** | **1061.5***** | 609 | **868.4***** | **813.7***** | **410.5*** | **800.2***** | **664.5***** |
|  | **[741.9 ; 1201.7]** | **[590.8 ; 960.0]** | **[608.3 ; 1514.7]** | [-249.6 ; 1467.5] | **[483.4 ; 1253.4]** | **[529.9 ; 1097.6]** | **[104.8 ; 716.3]** | **[416.1 ; 1184.3]** | **[469.2 ; 859.9]** |
| % of variation in ∆per capita health expenditure explained (R-squared) | 57.1 | 64.3 | 39.6 | 28.3 | 41.4 | 38.7 | 28 | 41 | 42.3 |
| F-statistic (Model df) | 22.4 (1) | 40.21 (1) | 9.736 (1) | 0.0226 (1) | 7.074 (1) | 5.36 (1) | 1.382 (1) | 5.727 (1) | 9.992 (1) |
| Model sig. | ******* | ******* | ******* | 0.8826 | ***** | ***** | 0.2581 | ***** | ***** |
| root MSE | 190.6 | 173.5 | 226.7 | 247 | 222.3 | 228.8 | 284.7 | 224.2 | 220.9 |
| Durbin-Watson statistic | 1.843 | 1.822 | 1.662 | 1.671 | 1.676 | 1.727 | 0.998 | 1.531 | 1.626 |
| N clusters | 16 | 16 | 16 | 16 | 16 | 16 | 16 | 16 | 16 |
|  |  |  |  |  |  |  |  |  |  |

95% confidence intervals in brackets; * p<0.05 ; ** p<0.01 ; *** p<0.001; calculated from robust standard errors, adjusted for N clusters. Estimates derived from univariate GLS linear regression models (Prais-Winsten-Regression). The category „Other“ comprises asylum-seekers with nationalities from Australia and Oceania, stateless asylum-seekers, and asylum-seekers for with unknown nationality.
