# Supplementary material for: Effect of Restricting Access to Health Care on Health Expenditures among Asylum-Seekers and Refugees: A Quasi-Experimental Study in Germany, 1994–2013
Source: PLoS One. 2015 Jul 22;10(7):e0131483. doi: 10.1371/journal.pone.0131483 (PMC4511805; doi:10.1371/journal.pone.0131483)
Supplement: S4 Table — 95% confidence intervals in brackets; * p<0.05; ** p<0.01; *** p<0.001; calculated from robust standard errors, adjusted for N clusters. Estimates derived from univariate GLS linear regression models (Prais-Winsten-Regression). The category, “Others”comprises asylum-seekers with nationalities from Australia and Oceania, stateless asylum-seekers, and asylum-seekers for with unknown nationality. (DOC) [file pone.0131483.s009.doc]

Table S4: Change in the attributable fraction among the exposed (AFe) per year or per one unit increase in ∆*NEEDt* (unadjusted estimates)

|  |  | **∆*NEED* variables** | | | | | | | |
| --- | --- | --- | --- | --- | --- | --- | --- | --- | --- |
|  | **Time** | **∆Age** | **∆Female** | **∆Non-institutional housing** | **∆Europe** | **∆Africa** | **∆America** | **∆Asia** | **∆Other** |
|  | (years) | (years) | (percentage-points) | | | | | | |
| Change in AFe (in %-points) per one unit increase in time/∆need | **-3.686**** | **4.221**** | **7.589***** | 0.655 | **1.837**** | **-4.126**** | 38.93 | **-2.135**** | **5.046***** |
|  | **[-5.940,-1.432]** | **[1.573,6.868]** | **[5.352,9.826]** | [-1.713,3.024] | **[0.646,3.027]** | **[-6.481,-1.771]** | [-53.62,131.5] | **[-3.443,-0.827]** | **[2.631,7.461]** |
| Intercept *(ß0)* | **80.25***** | **72.54***** | **47.87***** | 50.31 | **58.05***** | **56.61***** | **25.64*** | **53.68***** | **42.87***** |
|  | **[43.14,117.4]** | **[43.78,101.3]** | **[36.84,58.90]** | [-7.896,108.5] | **[37.96,78.14]** | **[41.91,71.31]** | **[5.946,45.33]** | **[33.65,73.71]** | **[32.44,53.29]** |
| % of variation in AFe explained (Adj. R-squared) | 54.1 | 39.9 | 60.7 | 24.4 | 42 | 44.5 | 19.2 | 42.4 | 51.6 |
| F-stat.(Model df) | 11.9 (1) | 11.55 (1) | 52.27 (1) | 0.348 (1) | 10.81 (1) | 13.95 (1) | 0.804 (1) | 12.1 (1) | 19.83 (1) |
| Model sig. | ** | ** | *** | 0.5641 | ** | ** | 0.3841 | ** | *** |
| root MSE | 16.24 | 14.05 | 11.37 | 15.78 | 13.71 | 13.55 | 18.84 | 13.81 | 12.61 |
| D-W statistic | 1.424 | 1.718 | 1.701 | 1.666 | 1.762 | 1.8 | 1.062 | 1.498 | 1.657 |
| N clusters | 18 | 16 | 16 | 16 | 16 | 16 | 16 | 16 | 16 |
|  |  |  |  |  |  |  |  |  |  |

95% confidence intervals in brackets; * p<0.05 ; ** p<0.01 ; *** p<0.001; calculated from robust standard errors, adjusted for N clusters. Estimates derived from univariate GLS linear regression models (Prais-Winsten-Regression). The category „Others“ comprises asylum-seekers with nationalities from Australia and Oceania, stateless asylum-seekers, and asylum-seekers for with unknown nationality.
