# Supplementary material for: Effect of Restricting Access to Health Care on Health Expenditures among Asylum-Seekers and Refugees: A Quasi-Experimental Study in Germany, 1994–2013
Source: PLoS One. 2015 Jul 22;10(7):e0131483. doi: 10.1371/journal.pone.0131483 (PMC4511805; doi:10.1371/journal.pone.0131483)
Supplement: S5 Table — 95% confidence intervals in brackets; * p<0.05; ** p<0.01; *** p<0.001; calculated from robust standard errors, adjusted for N clusters. Estimates derived from univariate GLS linear regression models (Prais-Winsten-Regression). The category, “Others”comprises asylum-seekers with nationalities from Australia and Oceania, stateless asylum-seekers, and asylum-seekers for with unknown nationality. (DOC) [file pone.0131483.s010.doc]

Table S1: Change in the attributable fraction among the exposed (AFe) per one unit increase in ∆*NEEDt* adjusted for secular trends, age and sex differences

|  | **M1** | **M2** | **M3** | **M4** | **M5** | **M6** | **M7** | **M8** | **M9** |
| --- | --- | --- | --- | --- | --- | --- | --- | --- | --- |
| Time | **-2.250***** | **-1.531***** | **-1.188*** | -0.859 | **-1.182*** | -1.287 | -1.051 | **-2.309*** | - |
|  | **[-2.889,-1.612]** | **[-2.241,-0.821]** | **[-2.186,-0.190]** | [-2.273,0.556] | **[-2.193,-0.172]** | [-3.186,0.613] | [-2.202,0.100] | **[-4.461,-0.157]** |  |
| **∆**Female | 1.594 | 0.327 | 1.908 | -3.676 | -0.433 | -3.617 | -3.764 | 2.015 | - |
|  | [-0.344,3.532] | [-2.546,3.201] | [-2.289,6.105] | [-10.64,3.291] | [-3.255,2.388] | [-11.15,3.919] | [-11.77,4.242] | [-3.010,7.041] |  |
| **∆**Age |  | 3.646 | **4.468*** | **6.198*** | **4.222**** | 5.805 | **4.623*** | **4.887*** | **8.186***** |
|  |  | [-0.131,7.422] | **[1.067,7.868]** | **[1.452,10.94]** | **[1.254,7.190]** | [-1.405,13.02] | **[0.0981,9.148]** | **[0.926,8.847]** | **[5.037,11.34]** |
| **∆**Non-institutional housing |  |  | -0.797 |  |  |  |  |  | **-1.670*** |
|  |  |  | [-1.886,0.291] |  |  |  |  |  | **[-3.084,-0.256]** |
| **∆**European |  |  |  | 1.916 |  |  |  |  | -0.031 |
|  |  |  |  | [-1.223,5.055] |  |  |  |  | [-3.638,3.576] |
| **∆**African |  |  |  |  | -1.016 |  |  |  | -1.147 |
|  |  |  |  |  | [-3.617,1.586] |  |  |  | [-5.510,3.216] |
| **∆**American |  |  |  |  |  | 9.062 |  |  | 7.719 |
|  |  |  |  |  |  | [-104.0,122.1] |  |  | [-53.19,68.63] |
| **∆**Asian |  |  |  |  |  |  | -2.234 |  | ‚- |
|  |  |  |  |  |  |  | [-6.737,2.270] |  |  |
| **∆**Other |  |  |  |  |  |  |  | -3.732 | - |
|  |  |  |  |  |  |  |  | [-12.27,4.809] |  |
| Intercept | **73.53***** | **60.70***** | **52.84***** | **47.28**** | **56.63***** | 22.83 | 41.42 | **80.16**** | 10.16 |
|  | **[51.85,95.21]** | **[32.23,89.17]** | **[27.82,77.86]** | **[15.33,79.23]** | **[29.85,83.40]** | [-50.28,95.93] | [-2.724,85.56] | **[28.90,131.4]** | [-35.41,55.74] |
| Adj. R-squared | 63.4 | 66.2 | 65.2 | 71.4 | 64.5 | 23.2 | 68.3 | 67.2 | 20.3 |
| F-stat. (Model dF) | 29.29 (2) | 53.75 (3) | 25.15 (4) | 54.36 (4) | 51.62 (4) | 35.23 (4) | 31.78 (4) | 24.06 (4) | 17.15 (5) |
| Model sig. | *** | *** | *** | *** | *** | *** | *** | *** | *** |
| root MSE | 10.97 | 10.59 | 10.78 | 9.848 | 10.89 | 17.48 | 10.35 | 10.56 | 17.45 |
| D-W-statistic | 1.85 | 1.866 | 1.873 | 2.008 | 1.918 | 1.331 | 1.888 | 1.864 | 1.44 |
| N | 16 | 16 | 16 | 16 | 16 | 17 | 16 | 16 | 17 |
|  |  |  |  |  |  |  |  |  |  |

95% confidence intervals in brackets; * p<0.05 ; ** p<0.01 ; *** p<0.001; calculated from robust standard errors, adjusted for N clusters. Estimates derived from univariate GLS linear regression models (Prais-Winsten-Regression). The category „Others“ comprises asylum-seekers with nationalities from Australia and Oceania, stateless asylum-seekers, and asylum-seekers for with unknown nationality.
